# LGR6 activates the Wnt/β-catenin signaling pathway and forms a β-catenin/TCF7L2/LGR6 feedback loop in LGR6^high^ cervical cancer stem cells

**DOI:** 10.1038/s41388-021-02002-1

**Published:** 2021-09-06

**Authors:** Qian Feng, Shan Li, Hong-Mei Ma, Wen-Ting Yang, Peng-Sheng Zheng

**Affiliations:** 1 Department of Reproductive Medicine, The First Affiliated Hospital of Xi’an Jiaotong University, Xi’an, 710061 Shaanxi People’s Republic of China; 2 Section of Cancer Stem Cell Research, Key Laboratory of Environment and Genes Related to Diseases, Ministry of Education of the People’s Republic of China, Xi’an, 710061 Shaanxi People’s Republic of China

**Keywords:** Cancer stem cells, Cervical cancer

## Abstract

The leucine-rich repeat-containing G-protein-coupled receptor 6 (LGR6) is considered to be a stem cell marker in many normal tissues and promotes tissue development, regeneration, and repair. LGR6 is also related to the initiation and progression of some malignant tumors. However, the role of LGR6 in cervical cancer has not been reported. Here, immunohistochemistry and western blotting showed that LGR6 was significantly upregulated in cervical cancer, compared with the normal cervix. By analyzing The Cancer Genome Atlas database, LGR6 was found to be correlated with a poor prognosis of cervical cancer. Then, a small population of LGR6^high^ cells isolated by using the fluorescence-activated cell sorting exhibited enhanced properties of cancer stem cells including self-renewal, differentiation, and tumorigenicity. Moreover, RNA sequencing revealed that LGR6 was correlated with the Wnt signaling pathway and TOP/FOP, reverse transcription-PCR, and western blotting further proved that LGR6 could activate the Wnt/β-catenin signaling pathway. Interestingly, LGR6 upregulated the expression of TCF7L2 by activating the Wnt/β-catenin pathway. Then, TCF7L2 combining with β-catenin in the nucleus enhanced LGR6 transcription by binding the promoter of LGR6, which further activated the Wnt signaling to form a positive feedback loop. Thus, our study demonstrated that LGR6 activated a novel β-catenin/TCF7L2/LGR6-positive feedback loop in LGR6^high^ cervical cancer stem cells (CSCs), which provided a new therapeutic strategy for targeting cervical CSCs to improve the prognosis of cervical cancer patients.

## Introduction

Cervical cancer is the fourth in female cancer with ~569,847 cases and 311,365 deaths in 2018 worldwide [1]. Vaccination and screening are effective for decreasing the prevalence rate of cervical cancer [2, 3]. Surgery, chemotherapy, or radiotherapy reduce the mortality of cervical cancer [4–6]. However, the prognosis of some women with recurrent or metastatic cervical cancer remains poor [7]. Cancer stem cells (CSCs), exhibiting stem cell characteristics including self-renewal, differentiation [8], and tumorigenic properties, play crucial roles in tumor initiation, metastasis, and recurrence [9]. Drugs targeting signaling pathways regulating CSCs could improve cancer patient prognosis [10, 11]. In recent studies, it has been found that some genes could play a role in maintaining CSC properties in cervical cancer. For example, *cPLA2α* could regulate different subsets of cervical CSCs [12]; *TET1* promotes stemness in cervical precancerous lesions [13]; *API5* facilitates cervical CSC-like properties [14]. Our previous studies have indicated that ALDH might be a marker of cervical CSCs [15] and some stem cell-associated genes such as *SOX2* [16], *OCT4* [17], and *LGR5* [18, 19] are closely associated with tumorigenesis in cervical cancer. Moreover, it has been reported that Erlotinib can overcome paclitaxel-resistant cervical CSCs [20] and zoledronic acid attenuates the stemness phenotype in cervical cancer by suppressing phosphorylated Erk1/2 and Akt [21].

The leucine-rich repeat-containing G-protein-coupled receptor 6 (LGR6) belongs to the G-protein-coupled receptor family, which marks stem cells in hair follicles [22], taste buds [23], lung [24, 25], nails [26], and mammary gland [27], and promotes tissue development, regeneration, and injury repair. In recent studies, LGR6 not only promotes the initiation and progression of cancers such as lung adenocarcinoma [28], luminal mammary tumor [27], and ovarian cancer [29] but also activates the phagocyte immunoresolvent function by binding to MaR1 [30]. LGR6 is closely related to LGR4 and LGR5. Interestingly, LGR4/5 tethered with Rnf43/Znrf3 by R-spondins forms the LGR–RSPOs–Rnf43/Znrf3 complex to prevent the ubiquitylation and degradation of Wnt receptors by Rnf43/Znrf3 and then activate the Wnt signaling pathway [31, 32]. Therefore, the LGR family is regarded as a coactivator for the Wnt signaling pathway. Furthermore, the Wnt pathway is crucial for the maintenance of CSC population [33, 34], and targeting Wnt signaling could potentially achieve antitumor effects [35]. Nevertheless, the role of LGR6 in cervical cancer is unknown.

Here, we revealed for the first time the role of LGR6 in cervical cancer. A small population of LGR6^high^ cervical cancer cells isolated by fluorescence-activated cell sorting (FACS) exhibited the property of CSCs including self-renewal, differentiation, and tumorigenicity. LGR6 activated a novel β-catenin/TCF7L2/LGR6-positive feedback loop in LGR6^high^ cervical CSCs.

## Results

### LGR6 is elevated in cervical cancer and is associated with a poor prognosis

**Fig. 1:**
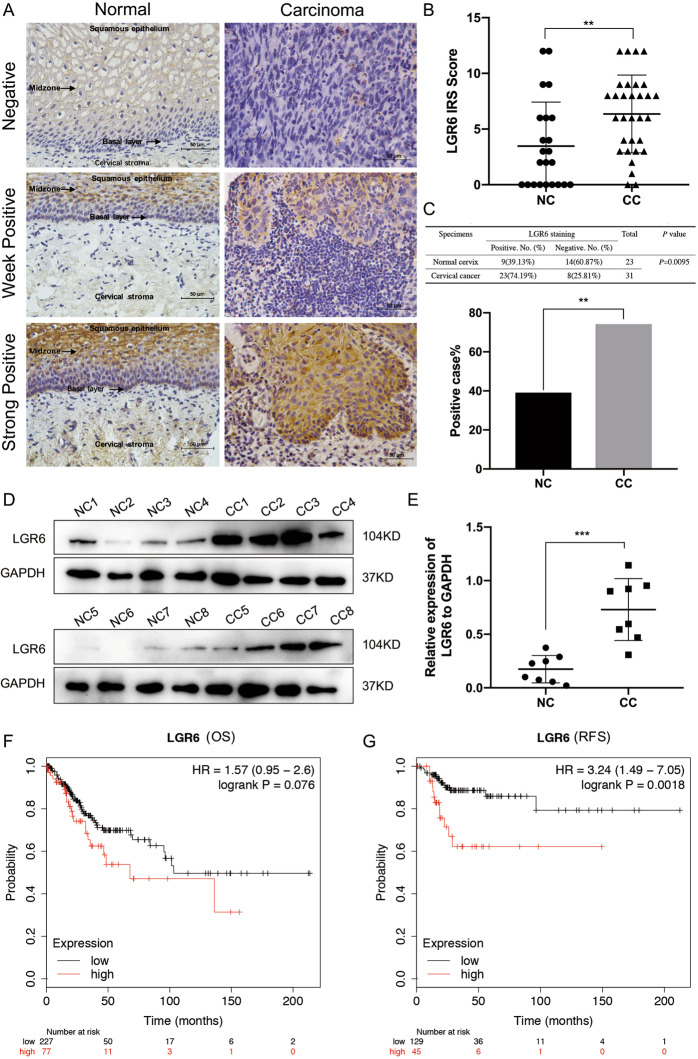
LGR6 is elevated in cervical cancer and associated with a poor prognosis. **A** The expression of LGR6 in normal cervical (NC) samples and cervical cell carcinoma (CC) samples were detected by Immunohistochemistry (IHC). The staining intensity of LGR6 was classified into three levels (negative, weak positive, and strong positive). **B** The scatter plots showed the IHC scores about the staining of LGR6 in NC and CC. Circle: NC, triangle: CC. Student’s *t* test was performed. **C** The correlation of LGR6 with cervical cancer was analyzed by *χ*^2^ test. The bar chart showed the percentage of LGR6-positive staining in NC (*n* = 23) and SCC (*n* = 31). **D**, **E** The levels of LGR6 in NC (*n* = 8) and CC (*n* = 8) samples were detected by western blotting. The quantitative analysis of LGR6/GAPDH in NC and SCC was shown (**e**). Circle: NC, square: CC. Student’s *t* test was performed. **F**, **G** The relationship between overall survival and relapse-free survival probability of cervical cancer patients (*n* = 304) and LGR6 in cervical cancer tumors were analyzed by Kaplan–Meier estimator in the TCGA database. **p < 0.01; ***p < 0.001.

To explore the role of LGR6 in cervical cancer, the subcellular location and expression of LGR6 in 31 cervical cancer and 23 normal cervix (NC) samples were detected by immunohistochemistry (IHC). Positive LGR6 staining was mainly localized to the cytoplasm and was discovered in 39.13% (9/23) of NC samples and in 74.19% (23/31) of cervical cancer samples. The IHC scores of LGR6 were 3.48 ± 3.94 in NC and 6.36 ± 3.49 in cervical cancer (Fig. 1A–C). Moreover, we used western blotting to test the expression of LGR6 in eight NC and eight cervical cancer tissues at random (Fig. 1D). The relative expression of LGR6 was 0.73 ± 0.29 in cervical cancer tissues and 0.17 ± 0.13 in NC tissues (Fig. 1E). Thus, LGR6 was upregulated in cervical cancer. Furthermore, we analyzed the overall survival (OS) and the relapse-free survival (RFS) probability in 304 patients with cervical cancer in The Cancer Genome Atlas (TCGA) RNA database by Kaplan–Meier plotter analysis (http://www.kmplot.com). The results demonstrated that although the level of LGR6 increased, the probability of cervical cancer patients’ RFS decreased with statistical significance (Fig. 1G, *p* = 0.0018). All data proved that LGR6 promoted cervical progression as a poor prognostic factor.

### Cervical cancer cells with high expression of LGR6 have enhanced self-renewal ability

**Fig. 2:**
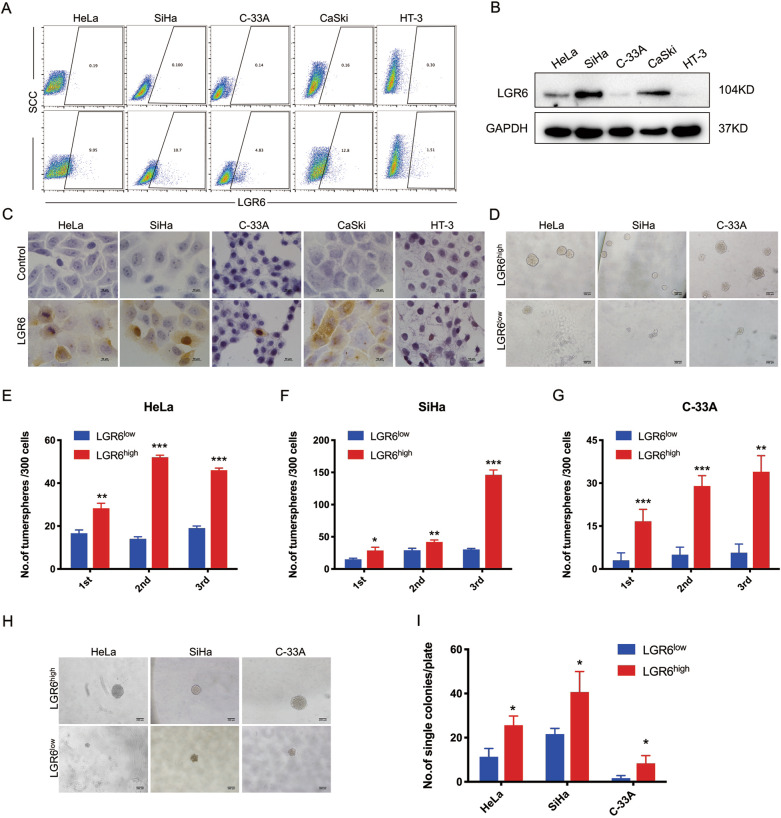
Cervical cancer cells with high expression of LGR6 have enhanced self-renewal ability. **A** LGR6 expression in five cervical cancer cell lines was detected by flow cytometry. Isotype control was that cells were treated with an isotype antibody. The gated cells represent the LGR6^high^ cells. **B** Western blotting (**B**) and ICC (**C**) showed the level of LGR6 in cervical cancer cell lines. **D**–**G** Representative photos of tumorspheres formed by LGR6^high^ and LGR6^low^ cells are shown on 24-well plates (**D**). The number of tumorspheres/300 cells from three consecutive passages was shown (**E**–**G**). **H**, **I** Representative photos of tumorspheres formed by LGR6^high^ and LGR6^low^ cells are shown on 96-well plates (**H**). The number of tumorspheres/1 cell was shown (**I**). *p < 0.05; **p < 0.01; ***p < 0.001. Data represent mean ± SD of triplicate experiments with Student’s *t* test.

Flow cytometry, western blotting, and immunocytochemistry (ICC) were used to detect the level of LGR6 in cervical cancer cell lines. Compared with the isotype control, LGR6 expression was found in 9.95% of HeLa cells, 10.7% of SiHa cells, 4.83% of C-33A cells, 12.8% of CaSki cells, 1.51% of HT-3 cells (Fig. 2A–C).

To assess the self-renewal ability of cervical cancer cells, LGR6^high^ and LGR6^low^ cells were cultured in a medium suitable for the growth of tumorspheres. LGR6^high^ cells from HeLa, SiHa, and C-33A cells generated more tumor spheroids than the LGR6^low^ cells did in the first passage, which was inoculated in 24-well plates with 300 cells per well. After consecutive subcultures for three passages, the populations of tumorsphere formed by the LGR6^high^ SiHa and C-33A cells gradually increased, while the LGR6^low^ SiHa and C-33A cells did not (Fig. 2D–G). To exclude the effects of cell aggregation, cells were inoculated in 96-well plates with a cell per well. LGR6^high^ cells from HeLa, SiHa, and C-33A cells generated more tumorspheres than the LGR6^low^ cells did (Fig. 2H, I; HeLa, 25.67 ± 4.163 vs 11.33 ± 3.78, *p* < 0.05; SiHa, 40.67 ± 9.29 vs 21.67 ± 2.51, *p* < 0.05; C-33A, 8.33 ± 3.51 vs 1.67 ± 1.15, *p* < 0.05). These findings demonstrated that LGR6^high^ cervical cancer cells had enhanced self-renewal potency compared with LGR6^low^ cervical cancer cells.

### Cervical cancer cells with high expression of LGR6 have enhanced tumorigenic potential in vivo

**Fig. 3:**
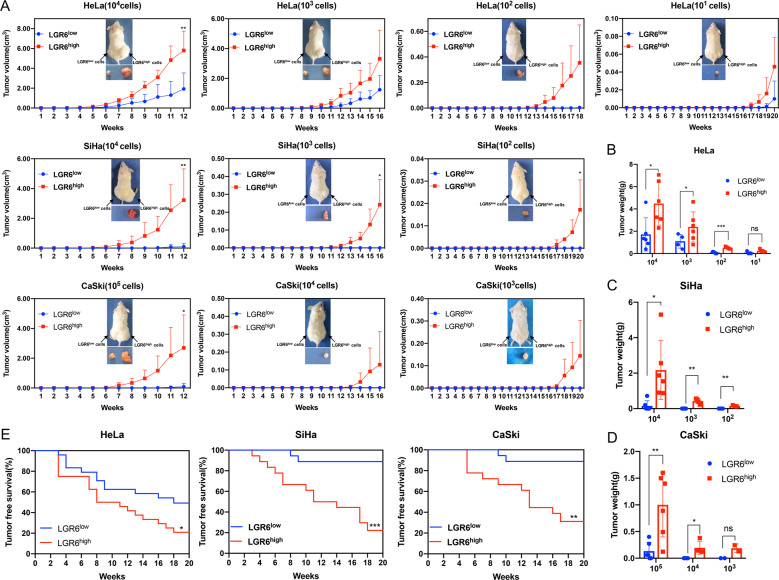
Cervical cancer cells with high expression of LGR6 have enhanced tumorigenic potential in vivo. **A** The volume of tumors formed by LGR6^high^ and LGR6^low^ cervical cancer cells was recorded (six mice per group). LGR6^high^ cervical cancer cells were injected into the right flank of NOD/SCID mice and LGR6^low^ cervical cancer cells were injected into the left flank of NOD/SCID mice. **B**–**D** Tumor weight was measured at the time of sacrifice. Data represent mean ± SD with Student’s *t* test. **E** Kaplan–Meier plots for tumor-free survival of mice after injection. Log-rank test was performed. n.s. not significant; *p < 0.05; **p < 0.01; ***p < 0.001.

To explore the function of LGR6 in tumorigenic potential, LGR6^high^ and LGR6^low^ cells separated from cervical cancer cells were injected into nonobese diabetic/severe combined immunodeficiency (NOD/SCID) mice by limiting dilution. Both LGR6^high^ and LGR6^low^ HeLa cells at doses of 10^4^, 10^3^, 10^2^, and 10^1^ cells generated tumors. Furthermore, the tumors formed by LGR6^high^ HeLa at a dose of 10^4^ cells were larger, heavier, and grew faster than those formed by LGR6^low^ cells (5.79 ± 1.95 vs 1.92 ± 1.61, *p* < 0.01; 4.48 ± 1.95 vs 1.72 ± 1.48, *p* < 0.05). The LGR6^high^ population from SiHa cells at doses of 10^3^ and 10^2^ cells and CaSki cells at doses of 10^4^ and 10^3^ cells, rather than the LGR6^low^ population, could form the palpable tumors. The tumors of LGR6^high^ cell from SiHa with the doses of 10^4^, 10^3^, and 10^2^ cells and CaSki cells at a dose of 10^5^ cells grew faster and heavier than those of LGR6^low^ cells (Fig. 3A–D).

Tumor latency was defined as the period of time when the mouse remained tumor-free. Here, LGR6^high^ HeLa cells had a lower tumor-free rate than LGR6^low^ HeLa cells (20.8% in LGR6^high^ cells vs 49.2% in LGR6^low^ cells, *p* < 0.05). Furthermore, LGR6^high^ SiHa cells showed a shorter tumor-free period (3 vs 8 weeks) and a lower tumor-free rate (22.2 vs 88.9%, *p* < 0.001) than LGR6^low^ SiHa cells. Similarly, LGR6^high^ CaSki cells also showed a shorter tumor-free period (5 vs 9 weeks) and a lower tumor-free rate (31.1 vs 88.9%, *p* < 0.01) than LGR6^low^ CaSki cells (Fig. 3E).

The CSC frequency of the LGR6^high^ and LGR6^low^ populations in three cervical cancer cell lines was calculated and shown in Table 1. LGR6^high^ HeLa cells had a stem cell frequency of 1/9.6, which was 5.9 times as high as that of LGR6^low^ HeLa cells (1/57.4; *p* < 0.001). Similarly, the stem cell frequency of LGR6^high^ SiHa cells (1/366) was 76.5 times higher than that of LGR6^low^ SiHa cells (1/28,004; *p* < 0.001). The stem cell frequency of LGR6^high^ CaSki cells (1/6696) was 16.3 times as high as that of LGR6^low^ CaSki cells (1/108,957; *p* < 0.001). All data proved that LGR6^high^ cervical cancer cells have greater tumorigenic potential than LGR6^low^ cervical cancer cells.

**Table 1:** Tumorigenic capacity of LGR6^high^ and LGR6^low^ cells in NOD/SCID mice from three cervical cancer lines.

Cell line	Subpopulation	Cell dose					Tumor-initiating cell frequency (95% interval)	*P* value
		10^5^	10^4^	10^3^	10^2^	10^1^		
HeLa	LGR6^high^	–	6/6	6/6	6/6	6/4	1/9.6 (1/3.78–1/25.9)	<0.0001
	LGR6^low^	–	6/6	6/4	6/2	6/1	1/57.4 (1/236.64–1/1380.5)	
SiHa	LGR6^high^	–	6/6	6/4	6/3	–	1/366 (1/146–1/1350)	<0.0001
	LGR6^low^	–	6/2	6/0	6/0	–	1/28,004 (1/7039–1/111,406)	
CaSki	LGR6^high^	6/6	6/4	6/2	–	–	1/6696 (1/2714–1/16523)	<0.0001
	LGR6^low^	6/4	6/0	6/0	–	–	1/108,957 (1/40,599–1/292,414)	

### Cervical cancer cells with high expression of LGR6 have the capability of differentiation in vitro and in vivo

**Fig. 4:**
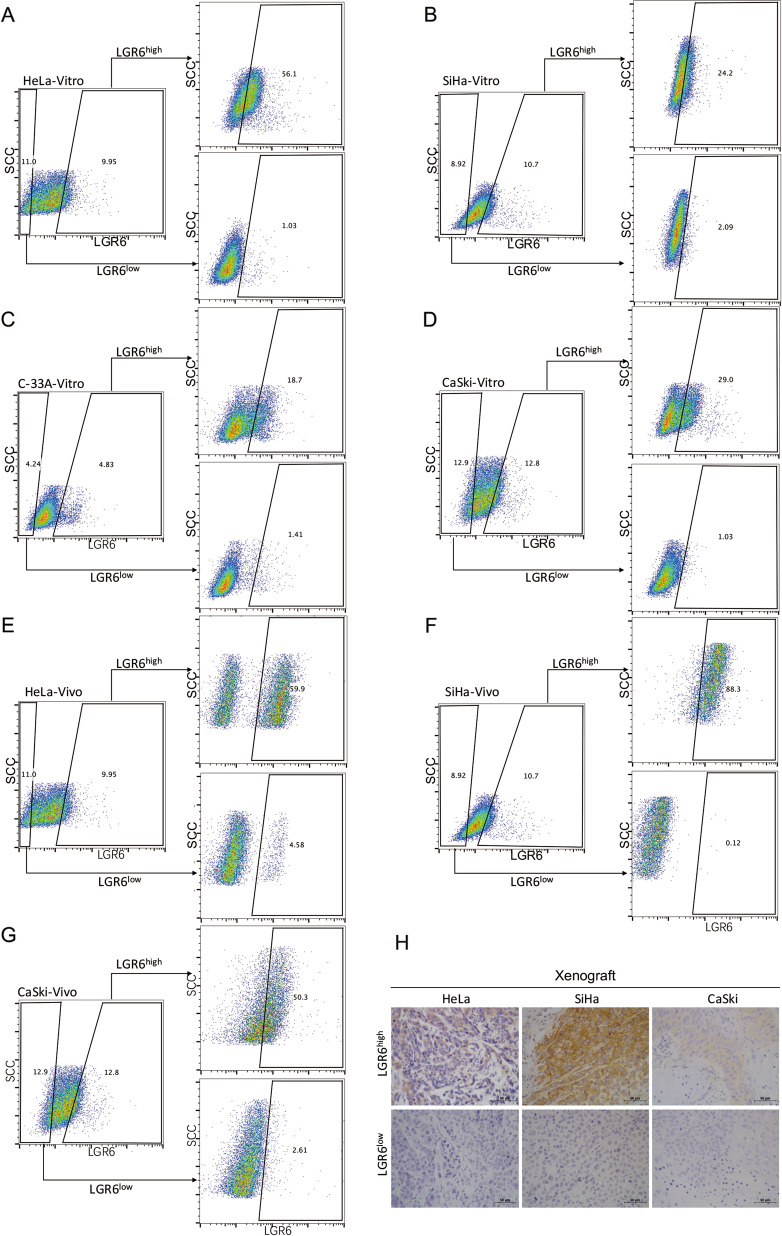
Cervical cancer cells with high expression of LGR6 have the capability of differentiation in vitro and in vivo. **A**–-**D** The expression of LGR6 in LGR6^high^ and LGR6^low^ cells from HeLa (**A**), SiHa (**B**), C-33A (**C**), or CaSki (**D**) cells cultured in DMEM medium supplemented with 10% FBS for 1 week was assessed by flow cytometry. The gated cells represent the LGR6^high^ cells. **E**–**H** The expression of LGR6 in the xenograft tumors formed by LGR6^high^ and LGR6^low^ cells was detected by flow cytometry (**E**–**G**) and IHC (**H**).

To explore whether LGR6^high^ cervical cancer cells have the ability to differentiate in vitro, LGR6^high^ and LGR6^low^ cells cultured separately in Dulbecco’s modified Eagle’s medium (DMEM) supplemented with 10% fetal bovine serum (FBS) for 1 week without passage were analyzed by flow cytometry. Approximately 43.9% of LGR6^high^ HeLa cells differentiated into LGR6^low^ HeLa cells, and 56.1% of these cells maintained the LGR6^high^ phenotype (Fig. 4A). Similarly, 75.8% of LGR6^high^ SiHa cells (Fig. 4B), 81.3% of LGR6^high^ C-33A cells (Fig. 4C), and 71% of LGR6^high^ CaSki cells (Fig. 4D) generated LGR6^low^ cells. Furthermore, 24.2% of LGR6^high^ SiHa cells, 18.7% of LGR6^high^ C-33A cells, and 29% of LGR6^high^ CaSki cells maintained the LGR6^high^ phenotype. In addition, ~98% of LGR6^low^ cells were still LGR6^low^ cells.

The differentiation ability of LGR6^high^ cells and LGR6^low^ cells was detected in vivo by flow cytometry and IHC. In the tumors generated by LGR6^high^ HeLa cells, 40.1% of LGR6^high^ HeLa cells differentiated into LGR6^low^ HeLa cells, indicating that LGR6^high^ HeLa cells could generate LGR6^high^ HeLa cells by the self-renewal ability and generate LGR6^low^ HeLa cells by the differentiation (Fig. 4E). Similarly, 11.7% of LGR6^high^ SiHa cells (Fig. 4F) and 49.7% of LGR6^high^ CaSki cells (Fig. 4G) generated LGR6^low^ cells. In addition, ~95% LGR6^low^ cells maintained the LGR6^low^ phenotype in vivo. Furthermore, we also used the IHC to assess the level of LGR6 in the LGR6^high^ and LGR6^low^ xenografts, which showed that some of LGR6^high^ cells did differentiate into LGR6^low^ cells (Fig. 4H). Therefore, a small population of LGR6^high^ cervical cancer cells established the cellular hierarchy by self-renewal and differentiation, which could be the cervical CSCs.

### LGR6 activates the Wnt signaling pathway and upregulates the stem cell-related factors in LGR6^high^ cervical CSCs

**Fig. 5:**
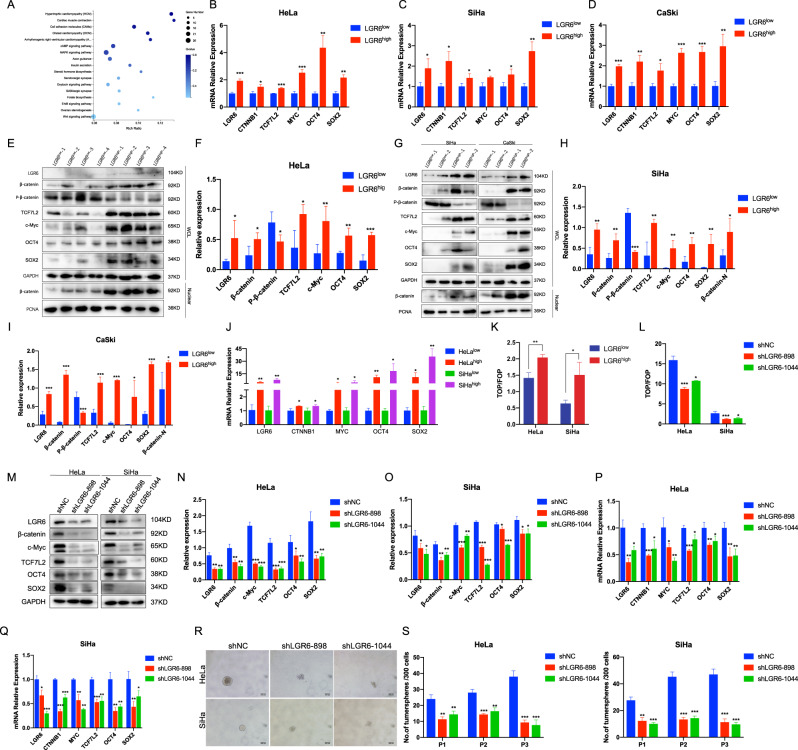
LGR6 activates the Wnt signaling pathway and upregulates the stem cell-related factors in LGR6^high^ cervical cancer stem cells. **A** Bubble chart of the KEGG pathway enrichment in RNA-sequencing data was shown. According to the KEGG pathway annotation and official classification, enrichment analysis of about 793 differentially expressed genes from RNA sequencing in the xenograft formed by LGR6^high^ HeLa cells and the xenograft formed by LGR6^low^ HeLa cells was done by the R software. The size of the bubble indicated the number of genes annotated to a certain KEGG pathway. The color represented the enriched *Q* value and the darker color represents the smaller the *Q* value. **B**–**I** The expression of Wnt signaling key genes and the pluripotency-associated stem cell factors in LGR6^high^ and LGR6^low^ xenograft tumor was detected by RT-PCR (**B**–**D**) and western blotting (**E**–**I**). The quantitative analysis of western blotting (**F**, **H**, **I**) was shown. **J** The mRNA level of *CTNNB1*, *MYC*, *OCT4*, and *SOX2* in LGR6^high^ and LGR6^low^ cells was tested by RT-PCR. **K**, **L** Cells sorted by LGR6 (**K**) and LGR6-silenced HeLa and SiHa (**L**) cells were transfected with the TOP/FOP-Flash reporter plasmids together with pRL-TK and the luciferase activities were measured 48 h after transfection. **M**–**Q** Western blotting (**M**) and RT-PCR (**P**, **Q**) were used to detect the Wnt signaling key genes and SOX2 and OCT4 expression in LGR6-silenced HeLa and SiHa cells. The quantitative analysis of western blotting was shown (**N**, **O**). **R**, **S** Representative photos of tumorspheres formed by shLGR6-898, shLGR6-1044, shNC HeLa, and SiHa cells are shown on 24-well plates (**R**). The number of tumorspheres/300 cells from three consecutive passages was shown (**S**). Data represent mean ± SD of triplicate experiments and were statistically analyzed with Student’s *t* test. *p < 0.05; **p < 0.01; ***p < 0.001.

To clarify how LGR6 worked in LGR6^high^ cervical CSCs, the gene expression profiles of tumor tissues formed by LGR6^high^ and LGR6^low^ HeLa cells were examined by RNA sequencing. A total of 17,735 transcripts were identified, of which 16,602 were commonly expressed in H1 (the xenograft formed by LGR6^high^ HeLa cells) and H2 (the xenograft formed by LGR6^low^ HeLa cells) including 793 differentially expressed genes (fold change ≥2.00 and false discovery rate ≤0.001) (Fig. S1a). Then, through Kyoto Encyclopedia of Genes and Genomes (KEGG) pathway enrichment analysis, several pathways related to stemness including the WNT, MAPK [36, 37], and ErbB signaling pathways [38] were exhibited (Fig. 5A). It has been reported that Lgr/R-spondin modules regulate the amplification of the Wnt signaling pathway [39]. Furthermore, LGR6 belonged to the LGR family, which could activate the Wnt signaling pathway in ovarian cancer [29]. Of these transcripts, Wnt signaling-related genes were further analyzed (Fig. S1b). We also analyzed the messenger RNA (mRNA) levels of some Wnt signaling-related genes in xenograft tumors from LGR6^high^ and LGR6^low^ HeLa, SiHa, and CaSki cells by reverse transcription-PCR (RT-PCR). The Wnt-driven *GREM1* [40] as a carcinogenic gene promoting proliferation in cervical cancer [41] and the Wnt target gene *AXIN2* [42] were upregulated in xenograft tumors from LGR6^high^ HeLa, SiHa, and CaSki cells. Moreover, LEF1 and LRP5 were also upregulated in xenograft tumors from LGR6^high^ HeLa and SiHa cells (Fig. S1c). Consistently, compared with xenograft tumors formed by LGR6^low^ cells, β-catenin and c-Myc levels were dominantly upregulated in the tumors generated by LGR6^high^ cells at the mRNA and protein levels (Fig. 5B–I and Fig. S2a–d). In addition, western blotting showed that β-catenin in the nucleus was increased and p-β-catenin (Ser33/37) was decreased in xenograft tumors formed by LGR6^high^ HeLa, SiHa, and CaSki cells compared with xenograft tumors formed by LGR6^low^ cells (Fig. 5E–I). It was reported that activated Wnt signaling could regulate the expression of the pluripotency-associated stem cell genes *OCT4* and *SOX2* [43–45]. Interestingly, *OCT4* and *SOX2* were also upregulated in LGR6^high^ HeLa xenograft tumors, as detected by RT-PCR (4.36 ± 0.89 vs 1 ± 0.14, *p* < 0.01; 2.17 ± 0.2 vs 1 ± 0.14, *p* < 0.01), western blotting (0.57 ± 0.13 vs 0.28 ± 0.03, *p* < 0.01; 0.57 ± 0.05 vs 0.15 ± 0.1, *p* < 0.001), and IHC, which were also confirmed in xenograft tumors from LGR6^high^ and LGR6^low^ SiHa and CaSki cells (Fig. 5G–I and Fig. S2a–d). Furthermore, the upregulation of *CTNNB1*, *MYC*, *SOX2*, and *OCT4* in LGR6^high^ cervical cancer cells was further validated by the RT-PCR assay in vitro (Fig. 5J).

To further prove that LGR6 activated the Wnt signaling in LGR6^high^ cervical cancer cells, the TOP/FOP-Flash luciferase reporter assay, which could reflect the activity of Wnt signaling, was used. LGR6^high^ HeLa and SiHa cells had the higher activity of TOP-Flash reporter than LGR6^low^ HeLa and SiHa cells (Fig. 5K, HeLa, 2.04 ± 0.09 vs 1.42 ± 0.16, *p* < 0.01; SiHa, 1.51 ± 0.38 vs 0.64 ± 0.1, *p* < 0.05). Furthermore, exogenous knockdown of LGR6 by short hairpin RNA, which has been verified by flow cytometry, ICC, and western blotting, dramatically inhibits TOP-Flash reporter activity in HeLa and SiHa cells (Fig. 5L–Q and Fig. S2e, f). Simultaneously, downregulation of CTNNB1, c-Myc, SOX2, and OCT4 was validated both at the mRNA and protein levels upon exogenous knockdown of LGR6 in HeLa and SiHa cells (Fig. 5M–Q). Moreover, LGR6 knockdown reduced the spheroid formation capacity of HeLa and SiHa cells (Fig. 5R, S). All these data suggested that LGR6 activated the Wnt signaling in LGR6^high^ cervical CSC.

To further verify the mechanism involving LGR6 in the Wnt signaling pathways, LGR6^high^ HeLa and SiHa cells were treated with XAV939 (a β-catenin inhibitor) [39]. LGR6^high^ HeLa and SiHa cells treated with XAV939 generated fewer tumorsphere than LGR6^high^ cells treated with dimethyl sulfoxide (DMSO) as the control (Fig. S3a). After consecutive subcultures for three passages, the tumorsphere formed by LGR6^high^ cells treated with XAV939 died, while the tumorspheres formed by LGR6^high^ cells treated with DMSO did not die (Fig. S3b). Furthermore, LGR6^high^ HeLa, SiHa, and CaSki cells were treated with XAV939 for 24 h at concentrations of 25, 50, and 100 μM, respectively. The expression of β-catenin, c-Myc, LGR6, and TCF7L2 protein in cells treated with XAV939 were decreased compared to those in the control cells (Fig. S3c–f). These findings demonstrated that XAV939 could inhibit the self-renewal ability of LGR6^high^ cervical cancer cells by inhibiting the activity of Wnt signaling.

Moreover, we also detected the mRNA levels of the other stem cell-related factors including KLF4, ALDH1A1, NANOG, LGR5, and LGR4 in xenograft tumors formed by LGR6^high^ and LGR6^low^ HeLa, SiHa, and CaSki cells. KLF4 and ALDH1A1 were upregulated in the tumors formed by LGR6^high^ HeLa and CaSki cells and LGR4 was downregulated in the tumors formed by LGR6^high^ SiHa and CaSki cells, compared with the tumors formed by LGR6^low^ cells (Fig. S4b–d). Simultaneously, the upregulations of KLF4 and ALDH1A1 and downregulation of LGR4 were validated at protein levels by western blotting (Fig. S4e–i) and IHC (Fig. S4j–m). To explore whether the key stem-like factors were lost as the differentiation of LGR6^high^ cells in vitro, we compared the mRNA levels of *LGR6*, *OCT4*, *SOX2*, *MYC*, and *KLF4* in HeLa and SiHa cells before FACS and LGR6^high^ HeLa and SiHa cells cultured in DMEM with 10% FBS for 1 week or 2 weeks after FACS. Compared with the LGR6^high^ HeLa and SiHa cells cultured for 1 week, LGR6 and the key stem-like factors were decreased in the cells cultures for 2 weeks (Fig. S4n, o). Therefore, the results illustrated that with the loss of LGR6 in vitro, some key stem-like factors were lost.

### TCF7L2 promotes LGR6 expression by directly binding to the promoter of LGR6 in cervical cancer

**Fig. 6:**
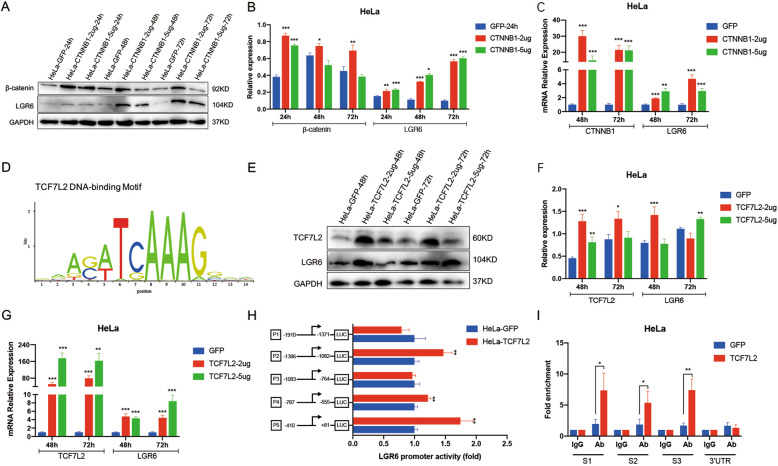
TCF7L2 promotes LGR6 expression by directly binding to the promoter of LGR6 in cervical cancer. **A**–**C** The expression of LGR6 and *CTNNB1* in *CTNNB1*-overexpressing and the control HeLa cells were detected by western blotting (**A**, **B**) and RT-PCR (**C**). **D** An experimentally defined transcription factor-binding sites of TCF7L2 were found in the JASPAR CORE database. **E**–**G** The expression levels of LGR6 and TCF7L2 in TCF7L2-overexpressing HeLa cells and the control cells were detected by western blotting (**E**, **F**) and RT-PCR (**G**). **H** The activity of the LGR6 promoter was detected by the dual-luciferase assay in TCF7L2-overexpressing and the control HeLa cells. **I** The qChIP in the TCF7L2-overexpressing and the control HeLa cells IP by TCF7L2 antibody and IgG antibody was displayed. Data represent mean ± SD of three independent experiments and were statistically analyzed with one-way ANOVA. *p < 0.05; **p < 0.01; ***p < 0.001.

We further explored the upstream regulatory molecules of LGR6. Fortunately, we accidentally discovered that overexpression of *CTNNB1* upregulated LGR6 expression in HeLa cells (Fig. 6A–C). We further proved that the Wnt agonist CHIR-99021 in HeLa and SiHa cells could promote the expression of LGR6 (Fig. S5a–d). Then, the ALGGEN (http://alggen.lsi.upc.es) databases predicted the transcription factors binding to the promoters of LGR6. TCF7L2 is the main transcription factor in the WNT signaling pathway [46]. The databases showed that TCF7L2 could bind the promoter of LGR6. Furthermore, the JASPAR (http://jaspar.genereg.net) and UCSC (http://genome.ucsc.edu/) databases predicted that the promoter region of LGR6 contains putative TCF7L2-binding sites (Fig. 6D). In further studies, western blot and RT-PCR demonstrated that overexpression of TCF7L2 upregulated LGR6 expression in cervical cancer cells at both the protein and mRNA levels (Fig. 6E–G), implying that TCF7L2 and β-catenin (*CTNNB1*) are upstream regulators of LGR6. These results were also confirmed in 293T cells (Fig. S6a–d). In addition, the dual-luciferase reporter assay was used. The LGR6 promoter region from −1910 to +81 (the LGR6 transcription start site is at 0) was divided into five segments containing the predicted TCF7L2-binding site. The data indicated that the luciferase activities of the P2 (−1386 to −1082), P4 (−787 to −555), and P5 (−410 to +81) promoters were increased in TCF7L2-overexpressing HeLa and 293T cells (Fig. 6H and Fig. S6e), which implied that the nucleotides −1386 to −1082, −787 to −555, and −410 to +81 in the LGR6 promoter may contain the TCF7L2-binding site. Then, we constructed three pairs of primers targeting the LGR6 promoter (Fig. S6f). The quantitative chromatin immunoprecipitation analysis showed that TCF7L2 bound to regions (−1295/1175, −759/661, and −306/196) of the LGR6 promoter in TCF7L2-overexpressing HeLa and 293T cells (Fig. 6I and Fig. S6g). These data demonstrated that TCF7L2 increased LGR6 transcription by directly binding to the promoter of LGR6.

### The function of LGR6 in primary cervical cancer cells from cervical cancer patients

Our data showed the role of LGR6 in cervical cancer cell lines. However, whether LGR6 possessed the same function in primary cervical cancers remained unknown. We cultured the primary cervical cancer cells derived from cervical cancer tissues (Fig. S7a). Three pairs of attached and spheroid cells of primary cervical cancer cells (P4, P7, and P9) were established (Fig. S7b). The mRNA levels of stem cell-related factors including NANOG, SOX2, MYC, and KLF4 were increased in the spheroid cells, compared with the attached cells. Simultaneously, LGR6 was also upregulated in the spheroid cells (Fig. S7c). The results demonstrated that the subpopulation of primary cervical cancer cells retained the self-renewal ability and LGR6 played a positive role in retaining the self-renewal ability of primary cervical cancer cells.

**Fig. 7:**
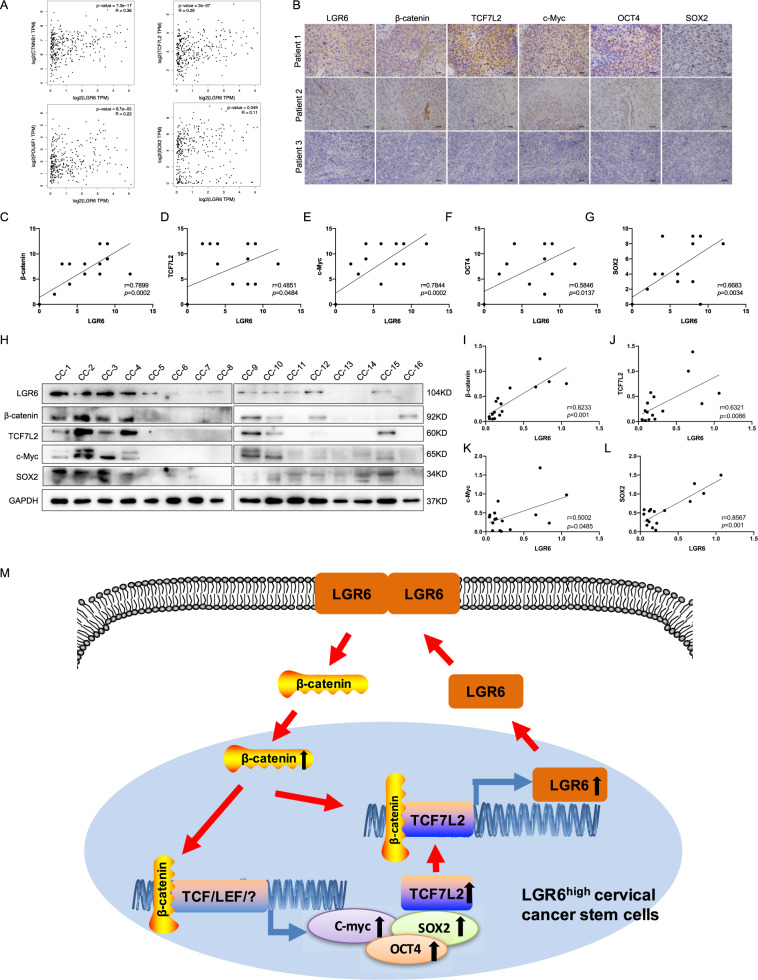
Clinical correlation between LGR6 and Wnt signaling-related genes in human cervical cancer tissues. **A** The corrections of *LGR6*, *CTNNB1*(β-catenin), *TCF7L2*, *POU5F1*(OCT4), *SOX2* in the TCGA CESC database. **B**–**G** LGR6, β-catenin, TCF7L2, c-Myc, OCT4, and SOX2 were detected by IHC in 15 cervical cancer samples. The representative images were shown (**B**). The correlation of the LGR6, β-catenin, TCF7L2, c-Myc, OCT4, and SOX2, respectively, was analyzed by Pearson’s test (**C**–**G**). **H**–**L** LGR6, β-catenin, TCF7L2, c-Myc, SOX2, and GAPDH were detected by western blotting in 16 cervical cancer samples (**H**). The correlation analysis was shown (**I**–**L**). **M** Schematic of LGR6 in cervical cancer stem cells. LGR6 increases the expression of β-catenin and promotes the nuclear translocation of β-catenin to activate the Wnt signaling and upregulate the expression of MYC, SOX2, OCT4, and TCF7L2. Then, increased transcription factor TCF7L2 combined with β-catenin in the nucleus further upregulates the expression of LGR6 by directly binding to the promoter of LGR6. LGR6 forms a novel β-catenin/TCF7L2/LGR6-positive feedback loop in a small proportion of LGR6^high^ cervical cancer stem cells.

Furthermore, we detected the expression of LGR6 in three primary cervical cancer cells (P4, P7, and P9 cells) by flow cytometry. Compared with the isotype control, LGR6 expression was found in 29.2% of P4 cells, 11.2% of P7 cells, and 45.8% of P9 cells (Fig. S7d). LGR6^high^ primary cervical cancer cells isolated by FACS could form tumor spheroids, while LGR6^low^ cells did not generate tumor spheroids (LGR6^low^ P4 and P9 cells) or form a few tumor spheroids (LGR6^low^ P7 cells), which disappeared after passaging (Fig. S7e and Fig. 7f). We also proved that LGR6^high^ commercial primary cervical cancer cells (PCC1 cells) could generate tumor spheroids (Fig. S7g–i). All data indicated that a rare population of LGR6^high^ primary cervical cancer cells had the self-renew ability.

### Clinical correlation between LGR6 and Wnt signaling-related genes in human cervical cancer tissues

According to the mechanism we explored above, we further proved the clinical correlation between LGR6 and Wnt signaling-related genes in cervical cancer patients. First, by analyzing the TCGA CESC database, we found that *LGR6* was positively correlated with *CTNNB1*, *TCF7L2*, *POU5F1*(OCT4), and *SOX2* at the mRNA levels (Fig. 7A). Then, we tested LGR6, β-catenin, TCF7L2, c-Myc, OCT4, and SOX2 in 15 cervical cancer tissues by IHC (Fig. 7B). β-Catenin, TCF7L2, c-Myc, OCT4, and SOX2 were positively correlated with LGR6 (Fig. 7C–G). Furthermore, we used western blotting to detect LGR6, β-catenin, TCF7L2, c-Myc, and SOX2 in 16 cervical cancer samples (Fig. 7H). Pearson’s correlation analysis also showed that LGR6 was positively correlated with β-catenin, TCF7L2, c-Myc and SOX2 (Fig. 7I-L). Interestingly, consistent with *LGR6*, *TCF7L2*, *CTNNB1*, *MYC*, and *POU5F1* were all related to the poor prognosis of cervical cancer (Fig. S8a–d).

## Discussion

CSCs play crucial roles in cancer progression, therapy resistance, and metastasis [47]. Therefore, treatment targeting CSCs might be an effective strategy to improve the prognosis of cervical cancer patients. In this study, we asserted that a small fraction of LGR6^high^ cervical cancer cells was strongly enriched for cervical CSCs and LGR6 activated the Wnt signaling in these LGR6^high^ cells.

First, bioinformatics and immunohistochemical assays supported that LGR6 served as an oncogene-promoting cervical cancer progression and functioned as a prognostic factor for the RFS of cervical cancer patients (Fig. 1). Then, LGR6^high^ cells isolated from HeLa, SiHa, and C-33A cells had enhanced spheroid formation capacity (Fig. 2). We observed that LGR6^high^ cervical cancer cells had the enhanced ability of tumorigenicity after LGR6^high^ and LGR6^low^ cells were injected into NOD/SCID mice (Fig. 3). Moreover, LGR6^high^ cells injected into NOD/SCID mice had a shorter tumor-free period, a lower tumor-free rate, and a higher CSC frequency than LGR6^low^ cells injected into NOD/SCID mice (Fig. 3e and Table 1). In addition, LGR6^high^ cells could re-establish the cellular hierarchy in vitro and in vivo (Fig. 4). In summary, this is the first time that LGR6 could mark a small population of cervical CSCs.

To further explore the mechanism by which LGR6 works in LGR6^high^ cervical CSCs, RNA sequencing of tumor tissues formed by LGR6^high^ and LGR6^low^ HeLa cells was performed (Fig. S1a). First, the KEGG pathway enrichment analysis in RNA sequencing showed that LGR6 was implicated in the regulation of Wnt signaling (Fig. 5A and Fig. S1b). Second, western blotting and RT-PCR showed that β-catenin was increased in LGR6^high^ cells by FACS (Fig. 5B–J). Knockdown of LGR6 led to the downregulation of β-catenin, c-Myc, SOX2, and OCT4 at both the mRNA and protein levels, suppression of TOP-Flash reporter activity, and spheroid formation capacity (Fig. 5L–S). Moreover, the TCGA database and our clinical specimens showed a positive correlation between LGR6 and CTNNB1 (β-catenin). It was reported that the Wnt signaling was an ancient and highly conserved pathway in stem cells and CSCs [48], and LGR6 marked CSCs and activated the Wnt pathway in lung adenocarcinoma [28]. In our study, LGR6 was proved for the first time to activate Wnt signaling in LGR6^high^ cervical CSCs. According to our previous studies, SOX2 and OCT4 play a crucial role in cervical cancer progression. Here, LGR6 is positively correlated with the expression of SOX2 and OCT4, which reveals that multiple genes form a stem cell network to regulate the characteristics of CCSCs and affect the progression and prognosis of cervical cancer.

TCF7L2 serves as a crucial transcription factor of the Wnt pathway mediating the downstream effects of Wnt signaling by interacting with β-catenin [46, 49]. First, we predicted that the promoter of LGR6 contained multiple binding sites of TCF7L2 based on UCSC and JASPAR databases (Fig. 6D). Second, by analyzing the TCGA database and our clinical specimens, we found that LGR6 was positively correlated with TCF7L2 in cervical cancer. Subsequently, the dual-luciferase assay and qChIP assay confirmed that TCF7L2 acted as a transcriptional activator to directly bind the promoter of LGR6 to induce the expression of LGR6 (Fig. 6H, I). Furthermore, TCF7L2 was also upregulated in LGR6^high^ cervical cancer cells. A previous study showed that β-catenin in the nucleus combined with SATB1 could upregulate the transcriptional level of TCF7L2 in colorectal cancer [50]. We proved that the Wnt agonist CHIR-99021 and overexpression of CTNNB1 could upregulate the expression of TCF7L2 in cervical cancer cells and 293T cells (Fig. S5). Therefore, LGR6 activated the Wnt signaling and upregulated the expression of TCF7L2 by increasing the expression of β-catenin and promoting the nuclear translocation of β-catenin. Then, transcription factor TCF7L2 combined with β-catenin in the nucleus further increased the expression of LGR6 (Fig. 7M). This is the first study to confirm that the existence of a novel β-catenin/TCF7L2/LGR6-positive feedback loop in cervical cancer, and LGR6 induces its own expression via the β-catenin/TCF7L2/LGR6 loop to further activate the Wnt signaling. Furthermore, as TCF7L2 expression increased, the probability of cervical cancer patients’ RFS decreased (Fig. S8a, *p* = 0.0054). Consistent with LGR6, TCF7L2 also predicted a poor prognosis in cervical cancer. Therefore, our research provided a theoretical basis for therapy targeting LGR6^high^ cervical CSCs to improve the prognosis of cervical cancer patients.

In summary, our study illuminated for the first time that LGR6 activated the Wnt/β-catenin signaling pathway and upregulated the protein level of pluripotency-associated stem cell factors including SOX2 and OCT4 in LGR6^high^ cervical CSCs. Furthermore, LGR6 was proven to increase its own expression through a novel β-catenin/TCF7L2/LGR6-positive feedback loop, which further activated the Wnt/β-catenin signaling pathway, and finally promoting cervical cancer progression and showing a vital role in a poor prognosis for cervical cancer patients.

## Materials and methods

### Cell lines, human tissue samples, and primary culture

The human cervical carcinoma cell lines (HeLa, SiHa, C-33A, CaSki, and HT-3) and 293T cells were bought from ATCC. HeLa, SiHa, C-33A, and 293T cells were grown in DMEM (Sigma-Aldrich); CaSki cells were grown in RPMI-1640 medium (Sigma-Aldrich); HT-3 cells were grown in McCoy’s 5A medium (Sigma-Aldrich). All mediums were supplemented with 10% FBS (Invitrogen, Carlsbad, CA, USA). NC and cervical cancer tissues were randomly obtained from the First Affiliated Hospital of Xi’an Jiaotong University, which was authorized by the human ethics committee. We excluded patients with chemotherapy, immunotherapy, or radiotherapy. The histological classifications and clinical staging were performed by the International Federation of Gynecology and Obstetrics classification system. Fresh cervical cancer tissues were obtained from nine cervical cancer patients after colpos biopsy or surgery used for the establishment of primary cervical cancer cells, which were authorized by the human ethics committee. The primary cervical cancer cells were cultured as described [51]. The primary cervical cancer cells (PCC1 cells) were bought from Fenghui Biotechnology Co., Ltd (Hunan province, China).

### IHC and ICC

The procedure of IHC and ICC staining was shown in the previous studies [52]. LGR6 staining was evaluated blindly and independently by two pathologists. The score for staining intensity (0 = no staining, 1 = light brown, 2 = brown, and 3 = dark brown) and staining frequency (0 = 0–10%, 1 = 11–25%, 2 = 26–50%, 3 = 51–75%, and 4 = 76–100%) were multiplied to obtain the immunoreactivity score (IRS). The IRS 0–3 was deemed negative, 4–6 weak positive, and >6 strong positive. Two different pathologists evaluated all the specimens in a blinded manner. The antibodies used were as follows: anti-LGR6 (#MAB8458, R&D Systems, USA), anti-β-catenin (#sc-7963, Santa Cruz, USA), anti-TCF7L2 (#sc-166699, Santa Cruz, USA), anti-c-Myc (#10828-1-AP, Proteintech, China), OCT4 (#sc-5279, Santa Cruz, USA), anti-SOX2 (#3579, Cell Signaling Technology, USA), anti-KLF4 (#sc-20691, Santa Cruz, USA), anti-ALDH1A1 (#sc-374149, Santa Cruz, USA), anti-LGR5 (#PAB2591, Abnova, Taiwan), anti-LGR4 (#sc-390630, Santa Cruz, USA).

### Statistical analysis

All data were analyzed by the SPSS 22.0 software (SPSS Inc., Chicago, IL, USA). The measurement data were shown as mean ± SD or SEM. The comparison between two groups was analyzed by two-tailed *t* test and among multiple groups were analyzed by *χ*^2^ test or one-way analysis of variance. The correlation of two genes was analyzed by Pearson’s test. The difference with *p* < 0.05 was considered statistically significant.

A detailed account was provided in the Supplementary section.

## Supplementary information

Supplement Figure 1

Supplement Figure 2

Supplement Figure 3

Supplement Figure 4

Supplement Figure 5

Supplement Figure 6

Supplement Figure 7

Supplement Figure 8

Supplement Table 1

Supplement figure legends and materials

Author Contribution Statement

## References

[1] Bray F, Ferlay J, Soerjomataram I, Siegel RL, Torre LA, Jemal A (2018). Global Cancer Statistics 2018: GLOBOCAN estimates of incidence and mortality worldwide for 36 cancers in 185 countries. CA Cancer J Clin.

[2] Brisson M, Kim JJ, Canfell K, Drolet M, Gingras G, Burger EA (2020). Impact of HPV vaccination and cervical screening on cervical cancer elimination: a comparative modelling analysis in 78 low-income and lower-middle-income countries. Lancet.

[3] Canfell K, Kim JJ, Brisson M, Keane A, Simms KT, Caruana M (2020). Mortality impact of achieving WHO cervical cancer elimination targets: a comparative modelling analysis in 78 low-income and lower-middle-income countries. Lancet.

[4] Rodin D, Burger EA, Atun R, Barton M, Gospodarowicz M, Grover S (2019). Scale-up of radiotherapy for cervical cancer in the era of human papillomavirus vaccination in low-income and middle-income countries: a model-based analysis of need and economic impact. Lancet Oncol..

[5] Höckel M, Wolf B, Schmidt K, Mende M, Aktas B, Kimmig R (2019). Surgical resection based on ontogenetic cancer field theory for cervical cancer: mature results from a single-centre, prospective, observational, cohort study. Lancet Oncol.

[6] da Costa SCS, Bonadio RC, Gabrielli FCG, Aranha AS, Dias Genta MLN, Miranda VC, et al. Neoadjuvant chemotherapy with cisplatin and gemcitabine followed by chemoradiation versus chemoradiation for locally advanced cervical cancer: a Randomized Phase II Trial. J Clin Oncol. 2019. 10.1200/JCO.19.00674. 10.1200/JCO.19.00674 31449470

[7] Cohen PA, Jhingran A, Oaknin A, Denny L (2019). Cervical cancer. Lancet.

[8] Viny AD, Bowman RL, Liu Y, Lavallée VP, Eisman SE, Xiao W (2019). Cohesin members Stag1 and Stag2 display distinct roles in chromatin accessibility and topological control of HSC self-renewal and differentiation. Cell Stem Cell.

[9] Nguyen LV, Vanner R, Dirks P, Eaves CJ (2012). Cancer stem cells: an evolving concept. Nat Rev Cancer.

[10] Visvader JE, Lindeman GJ (2012). Cancer stem cells: current status and evolving complexities. Cell Stem Cell.

[11] Clara JA, Monge C, Yang Y, Takebe N (2020). Targeting signalling pathways and the immune microenvironment of cancer stem cells - a clinical update. Nat Rev Clin Oncol.

[12] He Y, Xiao M, Fu H, Chen L, Qi L, Liu D, et al. cPLA2*α* reversibly regulates different subsets of cancer stem cells transformation in cervical cancer. Stem Cells. 2020. 10.1002/stem.3157 32100928

[13] Su PH, Hsu YW, Huang RL, Chen LY, Chao TK, Liao CC (2019). TET1 promotes 5hmC-dependent stemness, and inhibits a 5hmC-independent epithelial-mesenchymal transition, in cervical precancerous lesions. Cancer Lett.

[14] Song KH, Cho H, Kim S, Lee HJ, Oh SJ, Woo SR (2017). API5 confers cancer stem cell-like properties through the FGF2-NANOG axis. Oncogenesis.

[15] Liu SY, Zheng PS (2013). High aldehyde dehydrogenase activity identifies cancer stem cells in human cervical cancer. Oncotarget.

[16] Liu XF, Yang WT, Xu R, Liu JT, Zheng PS (2014). Cervical cancer cells with positive Sox2 expression exhibit the properties of cancer stem cells. PLoS ONE.

[17] Wang YD, Cai N, Wu XL, Cao HZ, Xie LL, Zheng PS (2013). OCT4 promotes tumorigenesis and inhibits apoptosis of cervical cancer cells by miR-125b/BAK1 pathway. Cell Death Dis.

[18] Chen Q, Cao HZ, Zheng PS (2014). LGR5 promotes the proliferation and tumor formation of cervical cancer cells through the Wnt/*β*-catenin signaling pathway. Oncotarget.

[19] Cao HZ, Liu XF, Yang WT, Chen Q, Zheng PS (2017). LGR5 promotes cancer stem cell traits and chemoresistance in cervical cancer. Cell Death Dis.

[20] Lv Y, Cang W, Li Q, Liao X, Zhan M, Deng H (2019). Erlotinib overcomes paclitaxel-resistant cancer stem cells by blocking the EGFR-CREB/GR*β*-IL-6 axis in MUC1-positive cervical cancer. Oncogenesis.

[21] Wang L, Liu Y, Zhou Y, Wang J, Tu L, Sun Z (2019). Zoledronic acid inhibits the growth of cancer stem cell derived from cervical cancer cell by attenuating their stemness phenotype and inducing apoptosis and cell cycle arrest through the Erk1/2 and Akt pathways. J Exp Clin Cancer Res.

[22] Snippert HJ, Haegebarth A, Kasper M, Jaks V, van Es JH, Barker N (2010). Lgr6 marks stem cells in the hair follicle that generate all cell lineages of the skin. Science.

[23] Ren W, Lewandowski BC, Watson J, Aihara E, Iwatsuki K, Bachmanov AA (2014). Single Lgr5- or Lgr6-expressing taste stem/progenitor cells generate taste bud cells ex vivo. Proc Natl Acad Sci USA.

[24] Ruiz EJ, Oeztuerk-Winder F, Ventura JJ (2014). A paracrine network regulates the cross-talk between human lung stem cells and the stroma. Nat Commun.

[25] Lee JH, Tammela T, Hofree M, Choi J, Marjanovic ND, Han S (2017). Anatomically and functionally distinct lung mesenchymal populations marked by Lgr5 and Lgr6. Cell.

[26] Lehoczky JA, Tabin CJ (2015). Lgr6 marks nail stem cells and is required for digit tip regeneration. Proc Natl Acad Sci USA.

[27] Blaas L, Pucci F, Messal HA, Andersson AB, Josue Ruiz E, Gerling M (2016). Lgr6 labels a rare population of mammary gland progenitor cells that are able to originate luminal mammary tumours. Nat Cell Biol.

[28] Guinot A, Oeztuerk-Winder F, Ventura JJ (2016). miR-17-92/p38alpha dysregulation enhances Wnt signaling and selects Lgr6+ cancer stem-like cells during lung adenocarcinoma progression. Cancer Res.

[29] Ruan X, Liu A, Zhong M, Wei J, Zhang W, Rong Y (2019). Silencing LGR6 attenuates stemness and chemoresistance via inhibiting Wnt/*β*-Catenin signaling in ovarian cancer. Mol Ther Oncolytics.

[30] Chiang N, Libreros S, Norris PC, de la Rosa X, Serhan CN (2019). Maresin 1 activates LGR6 receptor promoting phagocyte immunoresolvent functions. J Clin Invest.

[31] Carmon KS, Gong X, Lin Q, Thomas A, Liu Q (2011). R-spondins function as ligands of the orphan receptors LGR4 and LGR5 to regulate Wnt/beta-catenin signaling. Proc Natl Acad Sci USA.

[32] de Lau W, Peng WC, Gros P, Clevers H (2014). The R-spondin/Lgr5/Rnf43 module: regulator of Wnt signal strength. Genes Dev.

[33] Tammela T, Sanchez-Rivera FJ, Cetinbas NM, Wu K, Joshi NS, Helenius K (2017). A Wnt-producing niche drives proliferative potential and progression in lung adenocarcinoma. Nature.

[34] Vermeulen L, De Sousa EMF, van der Heijden M, Cameron K, de Jong JH, Borovski T (2010). Wnt activity defines colon cancer stem cells and is regulated by the microenvironment. Nat Cell Biol.

[35] Takebe N, Miele L, Harris PJ, Jeong W, Bando H, Kahn M (2015). Targeting Notch, Hedgehog, and Wnt pathways in cancer stem cells: clinical update. Nat Rev Clin Oncol.

[36] Day BW, Lathia JD, Bruce ZC, D’Souza RCJ, Baumgartner U, Ensbey KS (2019). The dystroglycan receptor maintains glioma stem cells in the vascular niche. Acta Neuropathol..

[37] Di Stefano B, Ueda M, Sabri S, Brumbaugh J, Huebner AJ, Sahakyan A (2018). Reduced MEK inhibition preserves genomic stability in naive human embryonic stem cells. Nat Methods..

[38] Powell AE, Wang Y, Li Y, Poulin EJ, Means AL, Washington MK (2012). The pan-ErbB negative regulator Lrig1 is an intestinal stem cell marker that functions as a tumor suppressor. Cell.

[39] Nusse R, Clevers H (2017). Wnt/*β*-catenin signaling, disease, and emerging therapeutic modalities. Cell.

[40] Lewis A, Freeman-Mills L, de la Calle-Mustienes E, Giráldez-Pérez RM, Davis H, Jaeger E (2014). A polymorphic enhancer near GREM1 influences bowel cancer risk through differential CDX2 and TCF7L2 binding. Cell Rep.

[41] Sun Q, Qi X, Zhang W, Li X (2021). Knockdown of circRNA_0007534 suppresses the tumorigenesis of cervical cancer via miR-206/GREM1 axis. Cancer Cell Int.

[42] Mazzoni SM, Fearon ER (2014). AXIN1 and AXIN2 variants in gastrointestinal cancers. Cancer Lett.

[43] Cui L, Guan Y, Qu Z, Zhang J, Liao B, Ma B (2013). WNT signaling determines tumorigenicity and function of ESC-derived retinal progenitors. J Clin Invest.

[44] Yi F, Pereira L, Hoffman JA, Shy BR, Yuen CM, Liu DR (2011). Opposing effects of Tcf3 and Tcf1 control Wnt stimulation of embryonic stem cell self-renewal. Nat Cell Biol.

[45] Yong X, Tang B, Xiao YF, Xie R, Qin Y, Luo G (2016). Helicobacter pylori upregulates Nanog and Oct4 via Wnt/*β*-catenin signaling pathway to promote cancer stem cell-like properties in human gastric cancer. Cancer Lett.

[46] Ravindranath A, O’Connell A, Johnston PG, El-Tanani MK (2008). The role of LEF/TCF factors in neoplastic transformation. Curr Mol Med.

[47] Lytle NK, Barber AG, Reya T (2018). Stem cell fate in cancer growth, progression and therapy resistance. Nat Rev Cancer.

[48] Valkenburg KC, Graveel CR, Zylstra-Diegel CR, Zhong Z, Williams BO (2011). Wnt/*β*-catenin signaling in normal and cancer stem cells. Cancers.

[49] Grove EA (2011). Wnt signaling meets internal dissent. Genes Dev.

[50] Mir R, Pradhan SJ, Patil P, Mulherkar R, Galande S (2016). Wnt/*β*-catenin signaling regulated SATB1 promotes colorectal cancer tumorigenesis and progression. Oncogene.

[51] Farias LM, Ocaña DB, Díaz L, Larrea F, Avila-Chávez E, Cadena A (2004). Ether a go-go potassium channels as human cervical cancer markers. Cancer Res.

[52] Li L, Yang WT, Zheng PS, Liu XF (2018). SOX17 restrains proliferation and tumor formation by down-regulating activity of the Wnt/*β*-catenin signaling pathway via trans-suppressing *β*-catenin in cervical cancer. Cell Death Dis.

