# Supplementary figures and images for: LGR6 activates the Wnt/β-catenin signaling pathway and forms a β-catenin/TCF7L2/LGR6 feedback loop in LGR6high cervical cancer stem cells
Source: Oncogene. 2021 Sep 6;40(42):6103–14. doi: 10.1038/s41388-021-02002-1 (PMC8530990; doi:10.1038/s41388-021-02002-1)

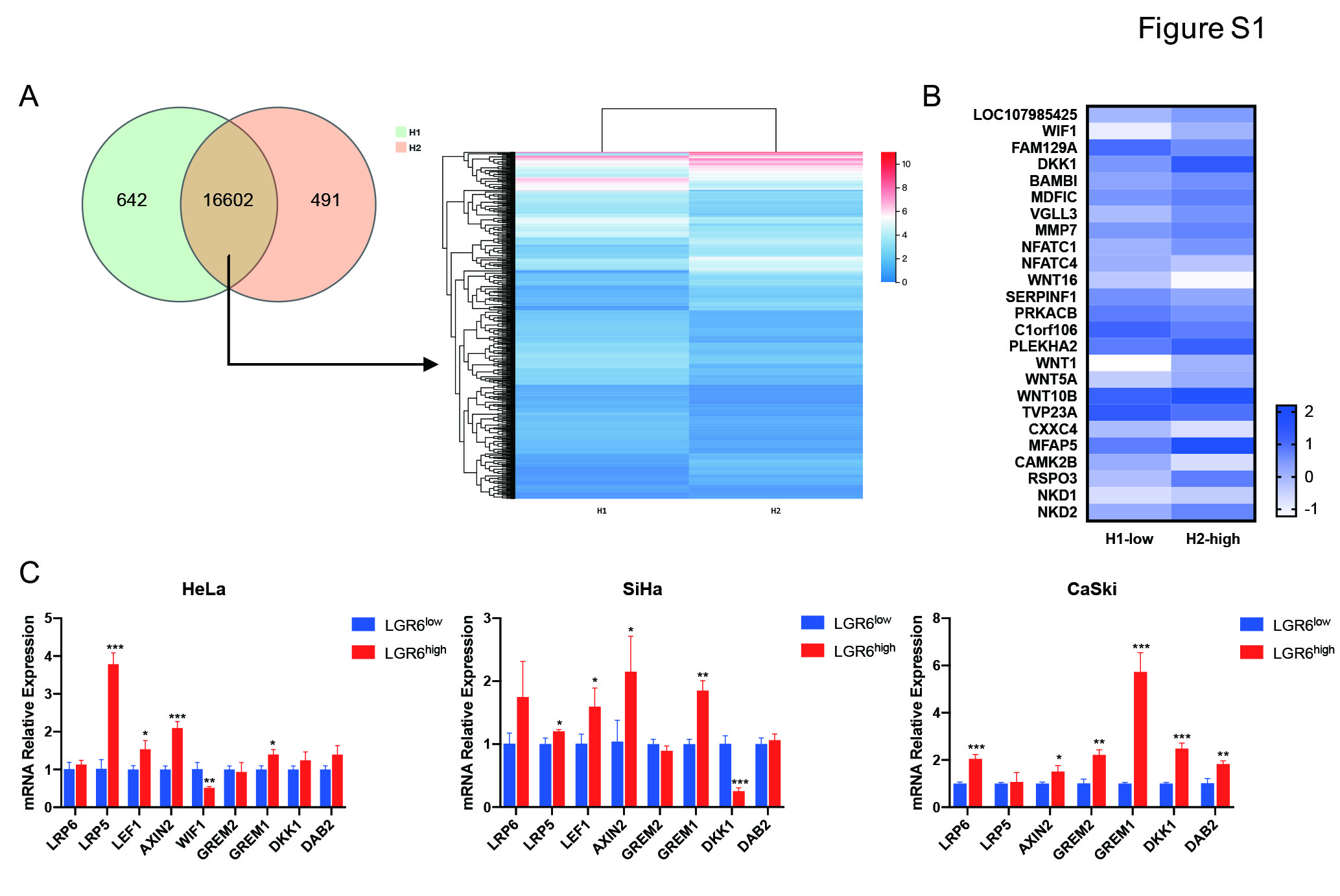

Supplement: Supplementary file 1 — Supplement Figure 1 [file 41388_2021_2002_MOESM1_ESM.jpg]

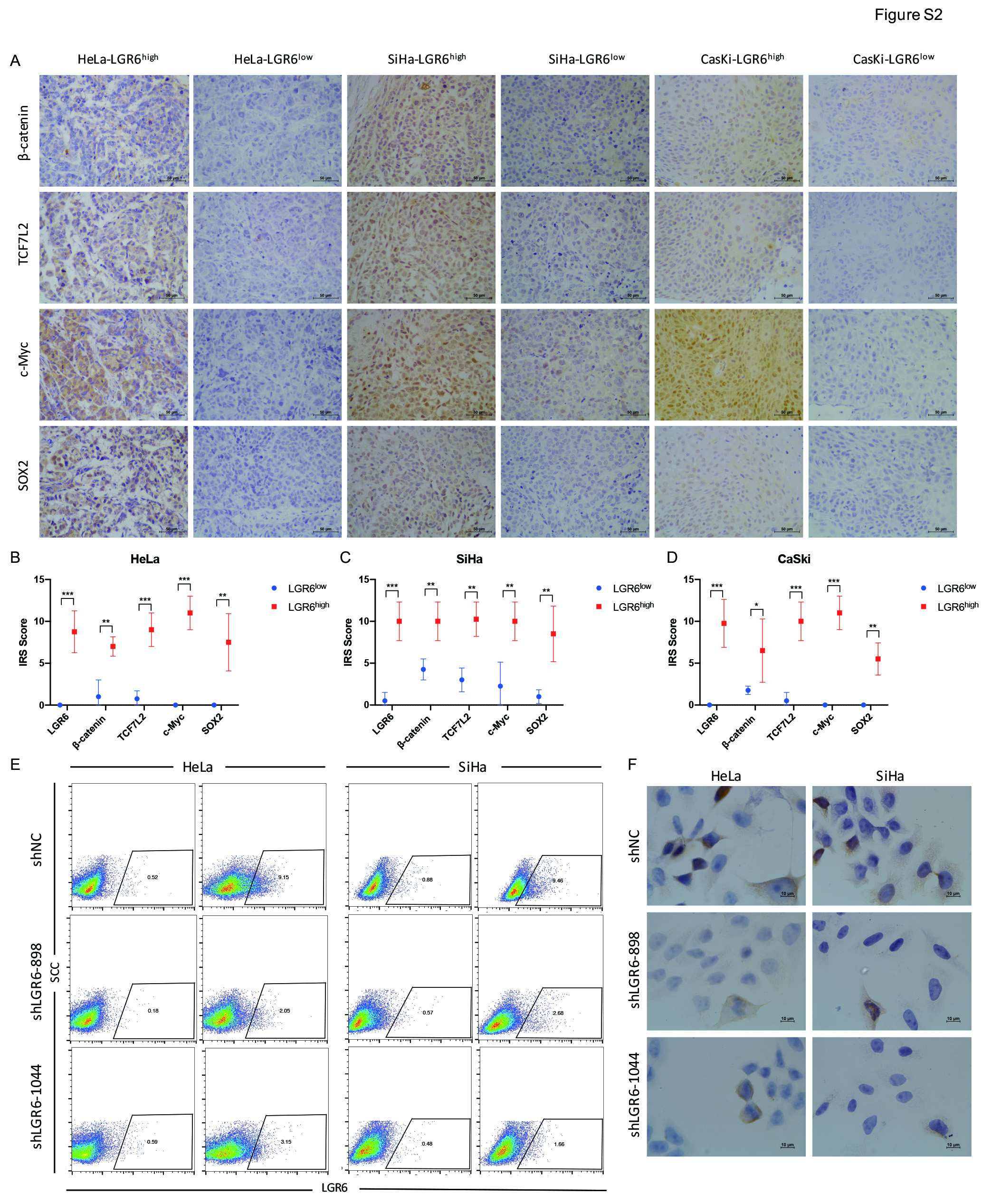

Supplement: Supplementary file 2 — Supplement Figure 2 [file 41388_2021_2002_MOESM2_ESM.jpg]

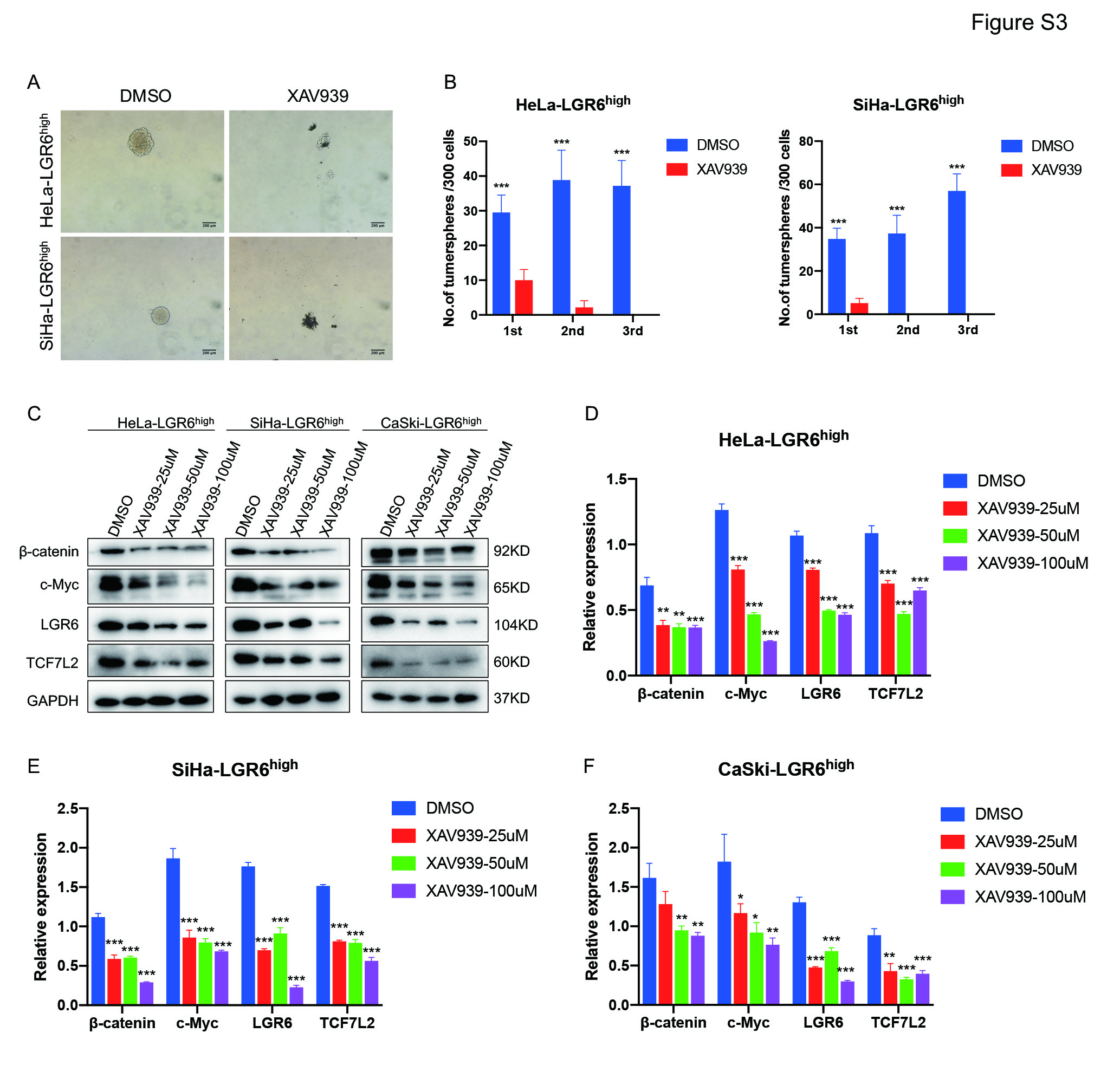

Supplement: Supplementary file 3 — Supplement Figure 3 [file 41388_2021_2002_MOESM3_ESM.jpg]

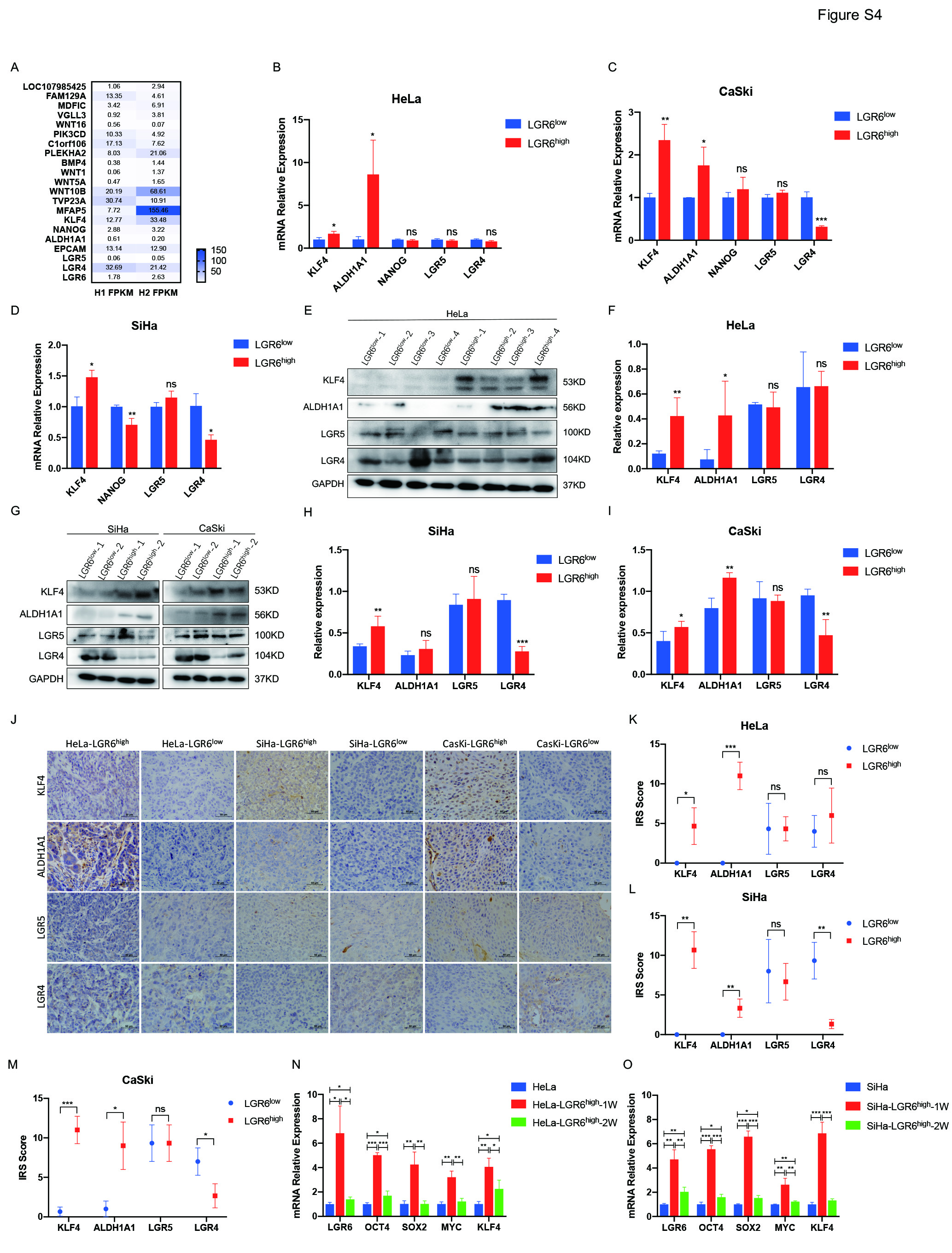

Supplement: Supplementary file 4 — Supplement Figure 4 [file 41388_2021_2002_MOESM4_ESM.jpg]

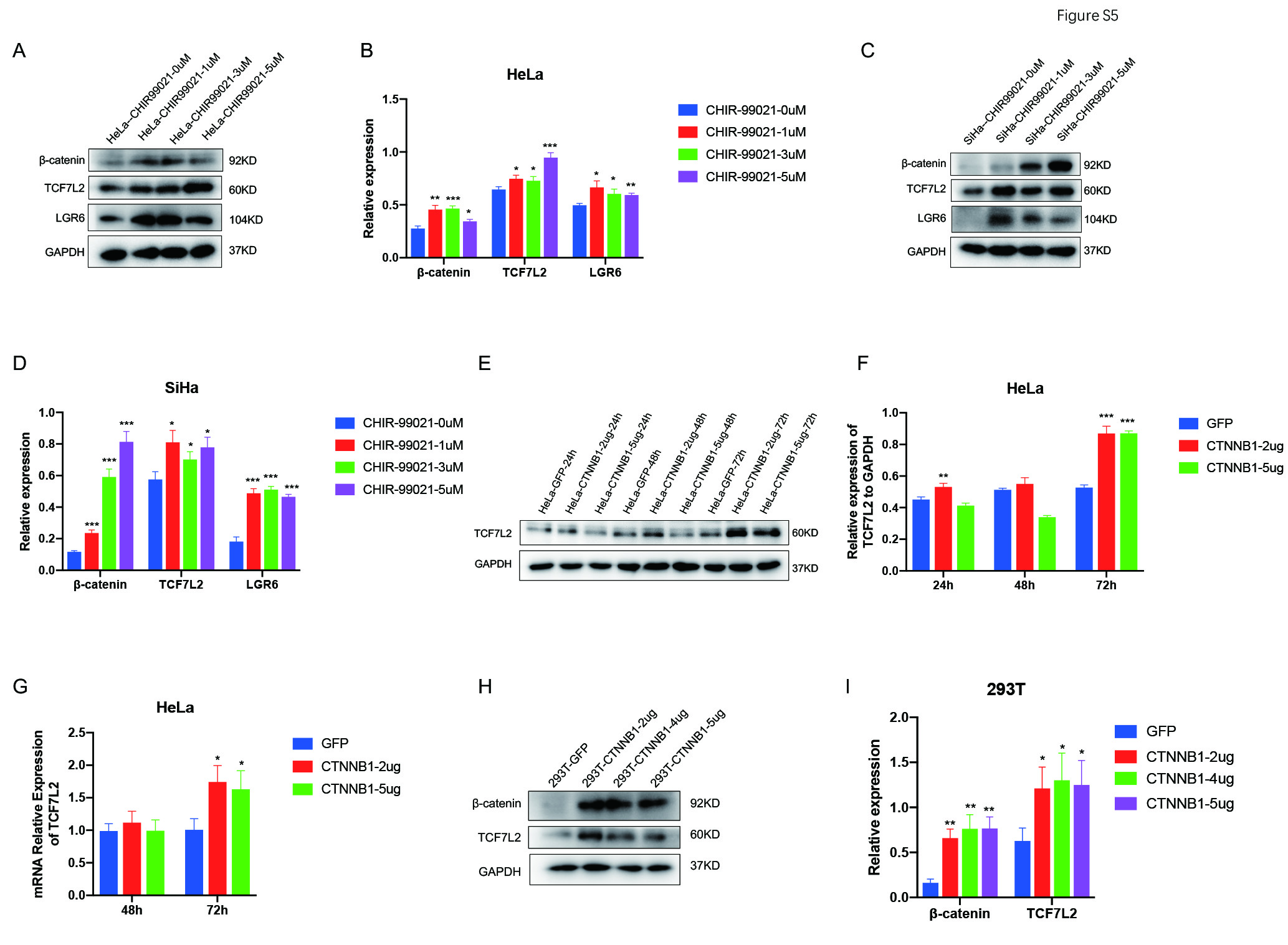

Supplement: Supplementary file 5 — Supplement Figure 5 [file 41388_2021_2002_MOESM5_ESM.jpg]

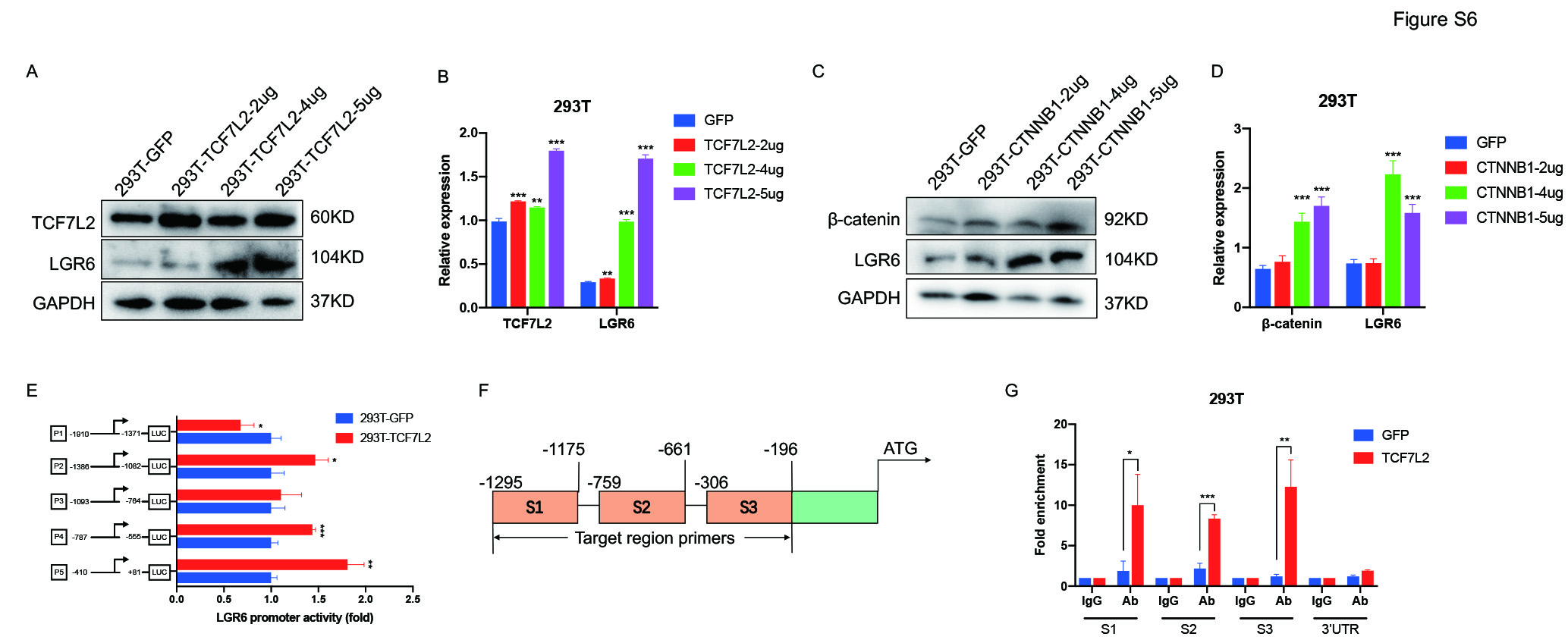

Supplement: Supplementary file 6 — Supplement Figure 6 [file 41388_2021_2002_MOESM6_ESM.jpg]

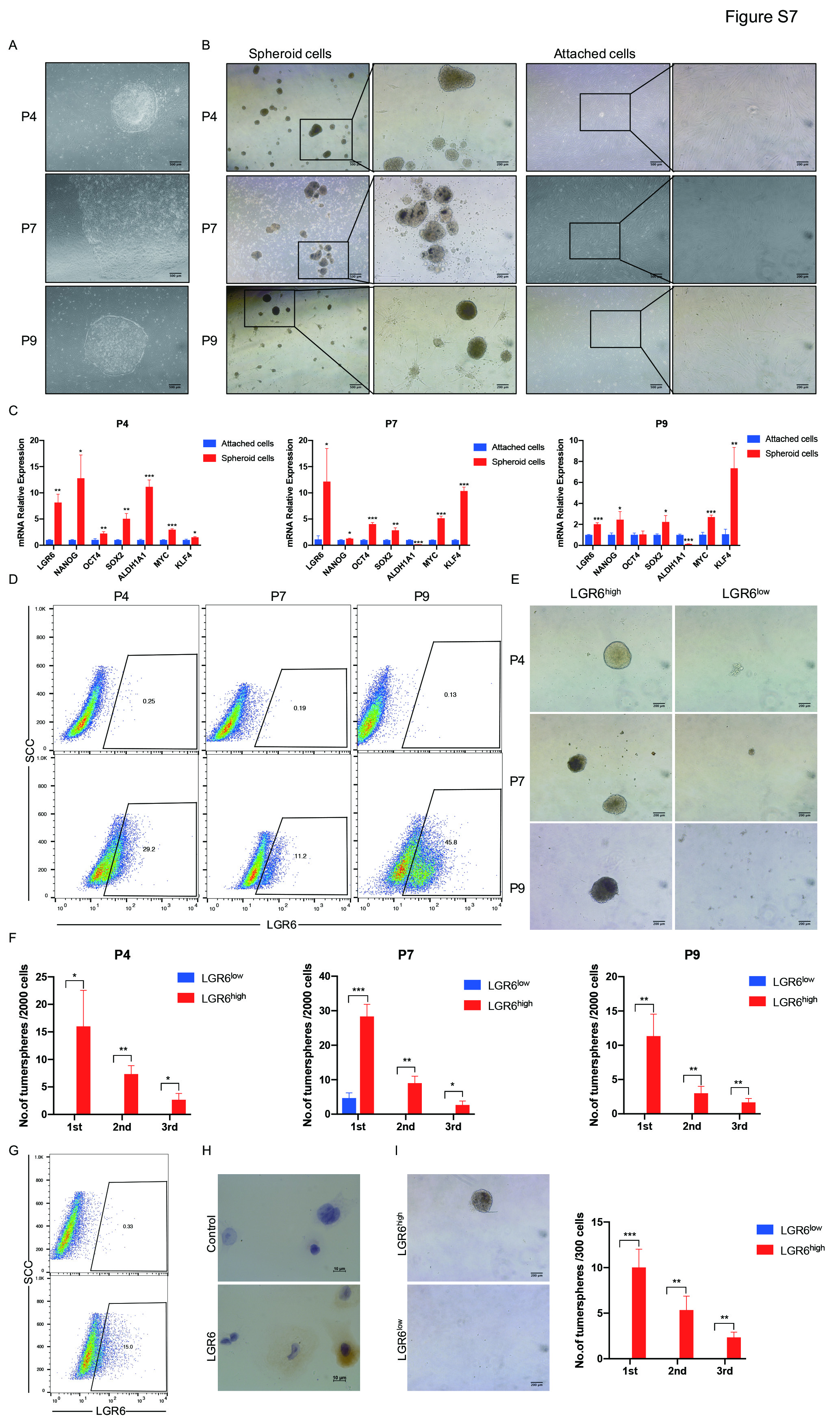

Supplement: Supplementary file 7 — Supplement Figure 7 [file 41388_2021_2002_MOESM7_ESM.jpg]

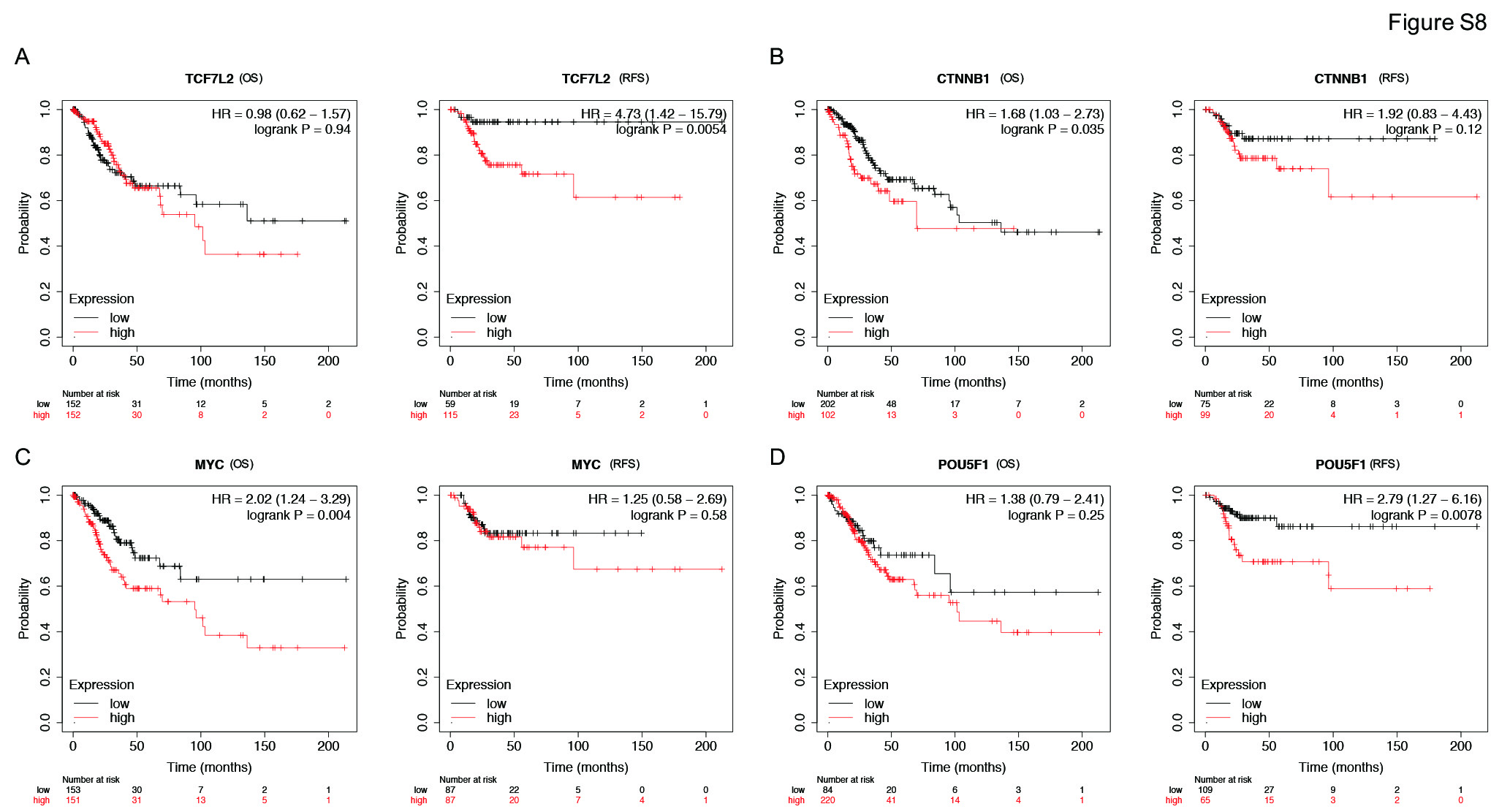

Supplement: Supplementary file 8 — Supplement Figure 8 [file 41388_2021_2002_MOESM8_ESM.jpg]
