# Supplementary material for: LGR6 activates the Wnt/β-catenin signaling pathway and forms a β-catenin/TCF7L2/LGR6 feedback loop in LGR6high cervical cancer stem cells
Source: Oncogene. 2021 Sep 6;40(42):6103–14. doi: 10.1038/s41388-021-02002-1 (PMC8530990; doi:10.1038/s41388-021-02002-1)
Supplement: Supplementary file 9 — Supplement Table 1 [file 41388_2021_2002_MOESM9_ESM.docx]

**Supplement Table 1: The list of primer sequences that used in this study**

| **Recombinant Vector Construction** | |
| --- | --- |
| TCF7L2-CDS | F: CCGGAATTCGCCACCATGCCGCAGCTGAACGG |
|  | R: CGCGGATCCCTATTCTAAAGACTTGGTGACGAGC |
| CTNNB1-CDS | F: ATGGCTACTCAAGCTGATTTGATG |
|  | R: TTACAGGTCAGTATCAAACCAGGC |
| shLGR6-898 | F:CACCGCATTCCAGTACCTGCCTAAATTCAAGAGATTTAGGCAGGTACTGGAATCCTTTTTTG |
|  | R:GATCCAAAAAAGCATTCCAGTACCTGCCTAAATCTCTTGAATTTAGGCAGGTACTGGAATGC |
| shLGR6-1071 | F:CACCGGAACTGTCTCACAATCAAATTTCAAGAGAATTTGATTGTGAGACAGTTCCTTTTTTG |
|  | R:GATCCAAAAAAGGAACTGTCTCACAATCAAATTCTCTTGAAATTTGATTGTGAGACAGTTCC |
| shLGR6-1448 | F:CACCGGATGTGTGCCAGCTTCTTCATTCAAGAGATGAAGAAGCTGGCACACATCCTTTTTTG |
|  | R:GATCCAAAAAAGGATGTGTGCCAGCTTCTTCATCTCTTGAATGAAGAAGCTGGCACACATCC |
| shLGR6-1044 | F:CACCGTGTCAGAAATTGGAGGAAATTTCAAGAGAATTTCCTCCAATTTCTGACACTTTTTTG |
|  | R:GATCCAAAAAAGTGTCAGAAATTGGAGGAAATTCTCTTGAAATTTCCTCCAATTTCTGACAC |
| shLGR6-1589 | F:CACCCCTGTGAGTACCTCTTTGAAATTCAAGAGATTTCAAAGAGGTACTCACAGGTTTTTTG |
|  | R:GATCCAAAAAACCTGTGAGTACCTCTTTGAAATCTCTTGAATTTCAAAGAGGTACTCACAGG |
| shLGR6-2172 | F:CACCCGGTGCCTACATCAAACTGTATTCAAGAGATACAGTTTGATGTAGGCACCGTTTTTTG |
|  | R:GATCCAAAAAACGGTGCCTACATCAAACTGTATCTCTTGAATACAGTTTGATGTAGGCACCG |
| shCtrl | F:CACCGTTCTCCGAACGTGTCACGTTTCAAGAGAACGTGACACGTTCGGAGAATTTTTTG |
|  | R:GATCCAAAAAATTCTCCGAACGTGTCACGTTCTCTTGAAACGTGACACGTTCGGAGAAC |
| **RT-PCR** | |
| LGR6 | F: TGGGGAACCCTCTGCTACAG |
|  | R: CAGGTACTGGAATGCCGATCT |
| TCF7L2 | F: AGAAACGAATCAAAACAGCTCCT |
|  | R: CGGGATTTGTCTCGGAAACTT |
| CTNNB1 | F: TCTGAGGACAAGCCACAAGATTACA |
|  | R: TGGGCACCAATATCAAGTCCAA |
| OCT4 | F: CTTGAATCCCGAATGGAAAGGG |
|  | R: GTGTATATCCCAGGGTGATCCTC |
| SOX2 | F: TACAGCATGTCCTACTCGCAG |
|  | R: GAGGAAGAGGTAACCACAGGG |
| MYC | F: CCTGGTGCTCCATGAGGAGA |
|  | R: TCCAGCAGAAGGTGATCCAGAC |
| KLF4 | F: CAGCTTCACCTATCCGATCCG |
|  | R: GACTCCCTGCCATAGAGGAGG |
| ALDH1A1 | F: GCACGCCAGACTTACCTGTC |
|  | R: CCTCCTCAGTTGCAGGATTAAAG |
| LGR5 | F: CACCTCCTACCTAGACCTCAGT |
|  | R: CGCAAGACGTAACTCCTCCAG |
| LGR4 | F: ACTCAAAGTTCTAACGCTCCAG |
|  | R: AAAGCACTCAGCCCTCGAATG |
| LRP5 | F: AACGGCAGGACGTGTAAGG |
|  | R: AGCGAGATCCTCCGTAGGTC |
| LGR6 | F: ACGATTGTAGTTGGAGGCTTG |
|  | R: ATGGCTTCTTCGCTGACATCA |
| LEF1 | F: AGAACACCCCGATGACGGA |
|  | R: GGCATCATTATGTACCCGGAAT |
| AXIN2 | F: CAACACCAGGCGGAACGAA |
|  | R: GCCCAATAAGGAGTGTAAGGACT |
| WIF1 | F: TCTCCAAACACCTCAAAATGCT |
|  | R: GACACTCGCAGATGCGTCT |
| GREM1 | F: TCATCAACCGCTTCTGTTACG |
|  | R: GGCTGTAGTTCAGGGCAGTT |
| GREM2 | F: ATCCCCTCGCCTTACAAGGA |
|  | R: TCTTGCACCAGTCACTCTTGA |
| DKK1 | F: CCTTGAACTCGGTTCTCAATTCC |
|  | R: CAATGGTCTGGTACTTATTCCCG |
| DAB2 | F: GTAGAAACAAGTGCAACCAATGG |
|  | R: GCCTTTGAACCTTGCTAAGAGA |
| NANOG | F: CCCCAGCCTTTACTCTTCCTA |
|  | R: CCAGGTTGAATTGTTCCAGGTC |
| GAPDH | F: GCACCGTCAAGGCTGAGAAC |
|  | R: TGGTGAAGACGCCAGTGGA |
| **Luciferase Assays** | |
| P1 (-1910 bp to-1371 bp) | F: CGACGCGTTCACTTTCCAGACTGGGCAGCCA |
|  | R: GGAAGATCTGAAAGTAGAAGACGAGCCTCCGCTC |
| P2 (-1386 bp to-1082 bp) | F: CGACGCGTCTCGTCTTCTACTTTCAGCGCCATG |
|  | R: GGAAGATCTGGTCTCTCCCATGCTGAGCCAA |
| P3 (-1093 bp to -764 bp) | F: CGACGCGTATGGGAGAGACCAGAGAGAGCAAGA |
|  | R: GGAAGATCTGCCTCTGTCAGGTTTACGGCCT |
| P4 (-787 bp to -555 bp) | F: CGACGCGTCCAGGCCGTAAACCTGACAGAG |
|  | R: GGAAGATCTCCTGGCCTGTCTGCTAGGACA |
| P5 (-410 bp to +81 bp) | F: CGACGCGTGAGACCTCCTCTGGGTCCCATGT |
|  | R: GGAAGATCTTCGGGCTACTGGGGCACCT |
| **Chromatin Immunoprecipitation Assay (ChIP)** | |
| S1 (-1295 bp— -1175 bp) | F: CTGGGAGGTTATTGGGAGAGTGG |
|  | R: AGCCAGAGGGTATACTAGAAACCTGG |
| S2 (-759 bp— -661 bp) | F: GAGTGCATTTCCACCCAGGGT |
|  | R: ACCAGGAGCAGTCTCCTCACTGA |
| S3 (-306 bp— -196 bp) | F: GTCTCTGACGGGCTGAGCTTTG |
|  | R: TCCTCTTCCCTCCTTCCTTCG |
| 3’UTR | F: ACAGTGAAGGGGTGGAGGGTT |
|  | R: GTATCCTATCCATGAGTCACACGGG |
