# Supplementary material for: LGR6 activates the Wnt/β-catenin signaling pathway and forms a β-catenin/TCF7L2/LGR6 feedback loop in LGR6high cervical cancer stem cells
Source: Oncogene. 2021 Sep 6;40(42):6103–14. doi: 10.1038/s41388-021-02002-1 (PMC8530990; doi:10.1038/s41388-021-02002-1)
Supplement: Supplementary file 10 — Supplement figure legends and materials [file 41388_2021_2002_MOESM10_ESM.doc]

**Material and Methods**

**Tumoursphere culture**

Cells were cultured in DMEM/F12 supplemented with 20 ng/mL human recombinant epidermal growth factor (#AF-100-15, Pepro Tech, USA) and 20 ng/mL basic fibroblastic growth factor (#100-18B, Pepro Tech, USA) together with N2 and B27 (#17502-048, #17504-044, Gibco) with 300 cells/well in 24-well plates or 1 cell/well in 96-well plates. The tumoursphere was observed within 2 weeks by two individuals blindly and independently. For serial tumoursphere formation assays, the spheres were collected, digested with 0.25% trypsin/EDTA, filtered by a 40 μm mesh and plated as described above. The experiments were repeated 3 times.

**Dual luciferase reporter assay**

TOP/FOP-Flash reporter and pTK-RL plasmids (#21-170, Millipore, Temecula, CA, USA) were used according to the instruction. Through predicted TCF7L2 binding site, the promoter of LGR6 was fused to pGL3-Basic Vector (#E1751, Promega, Madison, WI, USA). Cells were plated into 24-well plates and transfected with luciferase reporters vectors together with thymidine kinase promoter Renilla luciferase reporter plasmid (pRL-TK) and TCF7L2-overexpressing or control vector in triplicate by Lipofectamine 2000 (#11668019, Invitrogen, Carlsbad, CA, USA). Dual Luciferase Assay kit (#E1910, Promega) were used to detect the luciferase activity of cells 48 hours after transfection. The levels of promoter luciferase activity were normalized to Renilla luciferase activity. The primers are listed in Table S1.

**Plasmids and cell transfection**

Human TCF7L2 and β-catenin cDNA was amplified and subcloned into pIRES2-AcGFP (#632435, Clontech, Mountain View, CA) to form pIRES2-AcGFP-TCF7L2 and pIRES2-AcGFP-CTNNB1 plasmids (primers seen in Table S1). LGR6-specific short hairpin RNA (shRNA) with pGPU6/GFP/Neo was bought from GenePharma Co., Ltd (genepharma, Shanghai, China). Table S1 listed shRNA primers sequences used. Lipofectamine 2000 reagent (#11668019, Invitrogen, Carlsbad, CA, USA) were used to do the instantaneous transfection as the manufacturer’s instructions.

**Quantitative chromatin immunoprecipitation**

EZ-Magna ChIP Assay kit (#17-10086, Millipore, Darmstadt, Germany) were used to detect the binding sites of TCF7L2. We did it as the instructions. Anti-Histone H3 antibody (#4620, Cell Signaling Technology, USA), normal rabbit IgG (#2729, Cell Signaling Technology, USA) and TCF7L2 antibody (#2565, Cell Signaling Technology, USA) were used. The ChIP-enriched DNA fragments were detected and analyzed by RT-PCR. The primers used in qChIP were shown in Table S1.

**RNA resequencing**

The RNA of LGR6^high^ and LGR6^low^ HeLa xenograft tumour was extracted by RNAiso reagent (#9109, Takara, Osaka, Japan), which was detected by the BGISEQ-500 platform. Each sample contained 22.11 M data on average. The average rate of sample to genome was 88.08% and to gene set was 66.89%. The data was analyzed by PossionDis method, which identified differential expressed genes (DEGs) based on Fragments per Kilobase Million (FPKM)value fold change ≥2.0. The P-value was determined by False Discovery Rate(FDR) and FDR ≤ 0.001 was regarded as significant enrichment.

**Flow cytometry analysis and FACS**

The antibody of LGR6 (#MAB8458, RD System, USA) and second antibody (#A-21202, #12-4010-82, Invitrogen, USA) in accordance with manufacturer’s instructions were used for flow cytometry and FACS to measure the expression of LGR6 in human cervical cells and xenografts and sort LGR6^high^ cells and LGR6^low^ cells. Flow cytometry was performed with a FACSCalibu and FACS was performed with a BD FACSAria II cell sorter (Becton Dickinson). The data were analysed by FlowJo software (Tree Star Inc., Ashland, OR, USA). The xenograft tissues were minced and digested with 100 U/ml collagenase IV (#17104-019, Gibco, Grand Island, NY, USA) in basal medium at 37 °C overnight.

**In vivo tumour formation assays**

LGR6^high^ and LGR6^low^ HeLa, SiHa, CaSki cells sorted by FACS were resuspended in 200 μL 1:1 PBS/Matrigel (#356234, BD Biosciences) and injected subcutaneously into the left (LGR6^low^ cells) and right (LGR6^high^ cells) flanks of 6 to 8-week old female NOD/SCID mice (#406, Charles River Laboratory Animal Technology Co., Ltd., Beijing, China), which were observed twice per week. Eighteen mice were randomly divided into three groups each time, with six mice in each group. The tumour volume (V) was calculated by the length (a) and width (b) (V=ab^2^/2), which were presented as mean ± SEM .The frequency of stem cells was calculated by limiting-dilution analysis. The tumours tissues were collected, weighted, photographed and embedded in the paraffin.

**Western blotting**

The antibodies were as follows: anti-LGR6 (#ab126747, Abcam, USA), anti-β-catenin (#sc-7963, Santa Cruz, USA), anti-P-β-catenin (#sc-57535, Santa Cruz, USA), anti-TCF7L2 (#sc-166699, Santa Cruz, USA), anti-c-Myc (#10828-1-AP, Proteintech, China), anti-OCT4 (#sc-5279, Santa Cruz, USA), anti-SOX2 (#3579, Cell Signaling Technology, USA), anti-GAPDH (#sc-47724, Santa Cruz, USA), anti-KLF4 (#sc-20691, Santa Cruz, USA), anti-ALDH1A1 (#sc-374149, Santa Cruz, USA), anti-LGR5 (#PAB2591, Abnova, Taiwan), anti-LGR4 (#sc-390630, Santa Cruz, USA). And the second antibody were horseradish peroxidase-conjugated anti-rabbit or anti-mouse IgG (#G-21234, #G-21040, Thermo Fisher Scientific, New York, NY, USA).

**Supplement Figure Legends**

**FigureS1** **LGR6 is highly related to Wnt signaling in RNA sequencing.**

a Venn diagram illustration of LGR6^low^ xenograft tumour(H1) and LGR6^high^ xenograft tumour(H2) the overlap with expression genes determined by RNA sequencing. Heatmap of all differential gene expression between LGR6^high^ and LGR6^low^ xenograft tumour. b Heatmap of the differential gene expression in the Wnt signaling pathway between H1 and H2. Data were log10 normalized. c The expression of Wnt signaling key genes in LGR6^high^ and LGR6^low^ HeLa, SiHa, CaSki cells xenograft tumours was detected by RT-PCR. Data represent mean ± SD of triplicate experiments and were statistically analyzed with Student’s-test. * p < 0.05, ** p < 0.01, *** p < 0.001.

**FigureS2 LGR6 activates Wnt signaling in vivo.**

a-d The expression of β-catenin, TCF7L2, c-Myc and SOX2 in HeLa, SiHa and CasKi xenografts were detected by IHC. The representative pictures were shown(a). IRS scores were analyzed by Student’s-test (b-d). e LGR6 expression in shLGR6-898, shLGR6-1044, shNC HeLa and SiHa cells was detected by flow cytometry. Isotype control was that cells were treated with isotype antibody. The gated cells represent the LGR6^high^ cells. f ICC showed the level of LGR6 in shLGR6-898, shLGR6-1044, shNC HeLa and SiHa cells. * p < 0.05, ** p < 0.01, *** p < 0.001.

**FigureS3** **XAV939 inhibits the self-renewal ability of LGR6^high^ cervical cancer cells by inhibiting Wnt signaling.**

a Representative photos of tumourspheres formed by LGR6^high^ HeLa and SiHa cells cultured in medium suitable for the growth of tumourspheres and treated with XAV939 and DMSO are shown on 24-well plates. b The numbers of tumourspheres /300 cells from 3 consecutive passages were shown. c-f The expression of β-catenin, c-Myc, LGR6, TCF7L2 in LGR6^high^ HeLa, SiHa and CaSki cells treated with XAV939 for 24 hours at concentrations of 25μM, 50 μM and 100 μM was detected by western blotting (c). The quantitative analysis of western blotting (d-f) was shown. Data represent mean ± SD of triplicate experiments and were statistically analyzed with One-Way ANOVA. * p < 0.05, ** p < 0.01, *** p < 0.001.

**FigureS4 The stem cell-related factors were upregulated in LGR6^high^ cervical cancer cells.**

a Heatmap of the gene expression in the Signaling pathways regulating pluripotency of stem cells and LGR family between H1 and H2 by RNA sequencing. FPKM was shown. b-d The expression of KLF4, ALDH1A1, NANOG, LGR5, LGR4 in LGR6^high^ and LGR6^low^ xenograft tumours was detected by RT-PCR. e-m The expression of KLF4, ALDH1A1, LGR5 and LGR4 in LGR6^high^ and LGR6^low^ xenograft tumours was detected by western blotting (e, g) and IHC (j). The quantitative analysis of western blotting (f, h, i) and IHC(k-m) was shown. n, o The mRNA levels of LGR6, OCT4, SOX2, MYC, KLF4 in HeLa (n), SiHa (o) cells before fluorescence activated cell sorting (FACS) and LGR6^high^ HeLa (n), SiHa (o) cells cultured in DMEM with 10% FBS for one week or two weeks after FACS were shown. Data represent mean ± SD of triplicate experiments and were statistically analyzed with One-Way ANOVA. * p < 0.05, ** p < 0.01, *** p < 0.001.

**FigureS5 TCF7L2 is upregulated by CTNNB1 in cervical cancer cells and 293T cells.**

a-d The expression of TCF7L2, β-catenin and LGR6 in Hela (a) and SiHa (c) cells treated with CHIR99021 and DMSO were detected by western blotting. e-g The expression of TCF7L2 in CTNNB1-overexpressing and the control HeLa cells were detected by western blotting (e) and RT-PCR(g). h, i The expression of TCF7L2 in 293T-CTNNB1 and 293T-GFP cells were detected by western blotting (h). Data represent mean ± SD with One-Way ANOVA. * p < 0.05, ** p < 0.01, *** p < 0.001.

**FigureS6 TCF7L2 promotes the expression of LGR6 by binding to the promoter of LGR6 in 293T cells.**

a-d The expression levels of LGR6 in CTNNB1 and TCF7L2-overexpressing 293T cells and the control cells were detected by western blot. e The activity of the LGR6 promoter was measured by the dual luciferase assay in 293T-TCF7L2 and 293T-GFP cells. f The schematic diagram of the targeting regions of LGR6 promoter by TCF7L2. g The qChIP was shown in the TCF7L2-overexpressing and control 293T cells IP by TCF7L2 antibody and IgG antibody. Data represent mean ± SD with One-Way ANOVA. * p < 0.05, ** p < 0.01, *** p < 0.001.

**FigureS****7 The function of LGR6 in primary cervical cancer cells from cervical cancer patients.**

a Representative photos of primary cervical cancer cells. P4 cells were obtained from cervical cancer tissue of patient-4. P7 cells were obtained from cervical cancer tissue of patient-7. P9 cells were obtained from cervical cancer tissue of patient-9. b Representative photos of spheroid cells and attached cells formed by primary cervical cancer cells (P4, P7, P9 cells). c The levels of LGR6, NANOG, OCT4, SOX2, ALDH1A1, MYC, KLF4 were detected in spheroid cells and attached cells (P4, P7, P9 cells) by RT-qPCR. d LGR6 expression in primary cervical cancer cells (P4, P7, P9 cells) was detected by flow cytometry. Isotype control was that cells were treated with isotype antibody. The gated cells represented the LGR6^high^ cells. e, f Representative photos of tumourspheres formed by LGR6^high^ and LGR6^low^ primary cervical cancer cells (P4, P7, P9 cells) were shown on 24-well plates (e). The numbers of tumourspheres /2000 cells from 3 consecutive passages were shown (f). g, h LGR6 expression in commercial primary cervical cancer cells (PCC1 cells) was detected by flow cytometry(g) and ICC(h). i Representative photos of tumourspheres formed by LGR6^high^ and LGR6^low^ primary cervical cancer cells (PCC1 cells) were shown on 24-well plates. The numbers of tumourspheres /300 cells from 3 consecutive passages were shown. Data represent mean ± SD with Student’s-test. * p < 0.05, ** p < 0.01, *** p < 0.001.

**FigureS8** **TCF7L2, CTNNB1, MYC and POU5F1 are related to poor prognosis of cervical cancer.**

a-d The relationship between overall survival and relapse-free survival probability of cervical cancer patients (n = 304) and TCF7L2 (a), CTNNB1 (b), MYC (c), POU5F1 (d) were analysed by Kaplan–Meier estimator in the TCGA database.
