# Supplementary material for: LGR6 activates the Wnt/β-catenin signaling pathway and forms a β-catenin/TCF7L2/LGR6 feedback loop in LGR6high cervical cancer stem cells
Source: Oncogene. 2021 Sep 6;40(42):6103–14. doi: 10.1038/s41388-021-02002-1 (PMC8530990; doi:10.1038/s41388-021-02002-1)
Supplement: Supplementary file 11 — Author Contribution Statement [file 41388_2021_2002_MOESM11_ESM.docx]

**Author Contribution Statement**

Qian Feng designed and performed the experiments, analyzed the data, and wrote the manuscript. Shan Li performed a part of experiments and data analysis. Wen-Ting Yang and Hong-Mei Ma provided advice, helped with data analysis, and edited the manuscript. Peng-Sheng Zheng supervised the overall study, provided funding support, designed experiments, analyzed data, and wrote the manuscript. All authors read and approved the final paper.
